# Optimal Gearing of Musculoskeletal Systems

**DOI:** 10.1093/icb/icae072

**Published:** 2024-06-20

**Authors:** Delyle T Polet, David Labonte

**Affiliations:** Structure and Motion Lab, Royal Veterinary College, AL9 7TA, Hatfield, UK; Evolutionary Biomechanics Laboratory, Imperial College London, SW7 2AZ, London, UK

## Abstract

Movement is integral to animal life, and most animal movement is actuated by the same engine: striated muscle. Muscle input is typically mediated by skeletal elements, resulting in musculoskeletal systems that are geared: at any instant, the muscle force and velocity are related to the output force and velocity only via a proportionality constant *G*, the “mechanical advantage”. The functional analysis of such “simple machines” has traditionally centered around this instantaneous interpretation, such that a small vs large *G* is thought to reflect a fast vs forceful system, respectively. But evidence is mounting that a comprehensive analysis ought to also consider the mechanical energy output of a complete contraction. Here, we approach this task systematically, and deploy the theory of physiological similarity to study how gearing affects the flow of mechanical energy in a minimalist model of a musculoskeletal system. Gearing influences the flow of mechanical energy in two key ways: it can curtail muscle work output, because it determines the ratio between the characteristic muscle kinetic energy and work capacity; and it defines how each unit of muscle work is partitioned into different system energies, that is, into kinetic vs “parasitic” energy such as heat. As a consequence of both effects, delivering maximum work in minimum time and with maximum output speed generally requires a mechanical advantage of intermediate magnitude. This optimality condition can be expressed in terms of two dimensionless numbers that reflect the key geometric, physiological, and physical properties of the interrogated musculoskeletal system, and the environment in which the contraction takes place. Illustrative application to exemplar musculoskeletal systems predicts plausible mechanical advantages in disparate biomechanical scenarios, yields a speculative explanation for why gearing is typically used to attenuate the instantaneous force output ($G_{\text{opt}} < 1)$, and predicts how *G* needs to vary systematically with animal size to optimize the delivery of mechanical energy, in superficial agreement with empirical observations. A many-to-one mapping from musculoskeletal geometry to mechanical performance is identified, such that differences in *G* alone do not provide a reliable indicator for specialization for force vs speed—neither instantaneously, nor in terms of mechanical energy output. The energy framework presented here can be used to estimate an optimal mechanical advantage across variable muscle physiology, anatomy, mechanical environment, and animal size, and so facilitates investigation of the extent to which selection has made efficient use of gearing as a degree of freedom in musculoskeletal “design.”

## Introduction

*Give me a place to stand and with a lever I will move the whole world. -*Archimedes of Syracuse

Archimedes referred to them as “simple machines,” yet their influence on human culture, history, and technology has been anything but: cycling up a hill to get to work, building a pyramid to worship a supposed deity, or opening a cold drink on a hot summer’s day—many onerous tasks are substantially eased if not enabled by contrivances that provide a “mechanical advantage,” that is, that allow the movement of a large payload through application of a small input load. Machines that *leverage* a mechanical advantage are not just everywhere around us, but they are literally within us, for evolution has selected an engine that drives rotational movements by shortening: striated muscle.

Muscle is the primary animal motor, and it therefore stands to reason that understanding the limits to animal movement must involve an analysis of the physical and physiological constraints on muscle performance (Alexander 2003; Biewener and Patek 2018). As with Archimedes’s simple machines, a muscle’s force, shortening speed, and displacement are not, in general, equal to the force, speed, or displacement experienced by the object it actuates. Instead, muscle action is transmitted through joints, so that instantaneous muscle input and skeletal output are proportional to one another; musculoskeletal systems are generally geared. In modulating the effect of muscle input on skeletal output, gearing provides a degree of freedom on which evolutionary selection can act. But what, if any, is the best way to gear?

**Fig. 1:**
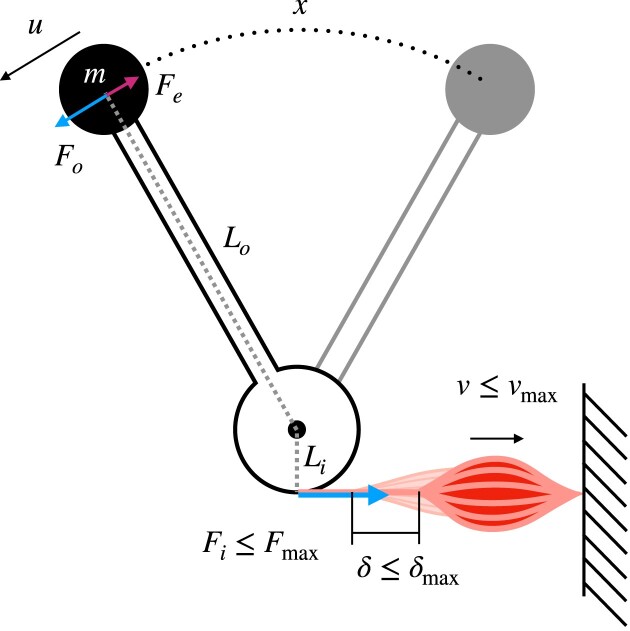
Schematic of the idealized musculoskeletal system studied in this paper. The muscle operates with constant mechanical advantage $G = L_i/L_o$, has a maximum contraction velocity $v_{\rm max}$, and a maximum displacement capacity $\delta _{\rm max}$. Once either limit is exceeded, muscle tensile force instantaneously drops from its maximum value $F_i(v \le v_{\rm max}, \delta \le \delta _{\rm max}) = F_{\text{max}}$ to zero, $F_i (v > v_{\rm max}, \delta > \delta _{\rm max}) = 0$. The inertia associated with the mass of the lever is assumed small relative to the inertia associated with the payload of mass *m*. External forces $F_e$ may resist motion, such that $F_{\text{net}} = F_i G - F_e$.

Musculoskeletal gearing manifests in many different forms: joints that connect skeletal segments (Borelli 1680; Gregory 1912; Gray 1944; Smith and Savage 1956); complex kinetic linkages formed of multiple rigid elements (Westneat 1994; Hulsey and Wainwright 2002; Anderson and Patek 2015; Olsen and Westneat 2016; Muñoz et al. 2017); muscle fiber pennation (Pfuhl 1937; Benninghoff and Rollhäuser 1952; Gans and Bock 1965; Brainerd and Azizi 2005); or, indeed, interlocking elements that look unmistakably like engineered gears (Burrows and Sutton 2013). Irrespective of the implementation of gearing, its effect can be described through the introduction of a mechanical advantage, *G*, usually defined by the ratio of two moment or lever arms, $L.$ For a simple hinge joint (Fig. 1), it is convenient to define these lever arms as the perpendicular distances between the joint axis of rotation and the lines of action of the input and output force vectors ($L_i$ and $L_o$, respectively; Fig. 1), so that the rotational dynamics can be written as a scalar expression. The proportionality constants that link instantaneous output ($F_o$) and input force ($F_i$), instantaneous input (*v*) and output velocity (*u*), and input ($\delta$) and output displacement (*x*) are then equal to the ratio between an effective in-lever ($L_i$) and an effective out-lever ($L_o$):


(1) \begin{eqnarray*}
\frac{F_o}{F_i} = \frac{L_i}{L_o} = G_F,
\end{eqnarray*}

(2) \begin{eqnarray*}
\frac{u}{v} = \frac{L_o}{L_i} = G_v,
\end{eqnarray*}

(3) \begin{eqnarray*}
\frac{x}{\delta } = \frac{L_o}{L_i} = G_v.
\end{eqnarray*}

Here, we make the common assumption that the lever itself is of negligible mass, and note that the same musculoskeletal system can operate with different $G_i$, for example, by altering the position of food along a jaw and the jaw opening angle. The velocity (or displacement) advantage, $G_v = L_o L_i^{-1}$ is used by engineers to parameterize gears and appears occasionally in the biomechanical literature (e.g. Carrier et al. 1998; Azizi and Brainerd 2007; McHenry 2012); the force advantage, $G_F = L_i L_o^{-1}$, in turn, is more common in the biomechanical literature, perhaps because of its historical origin in the work of Borelli (1680). For convenience, one of the two ratios is typically referred to as the mechanical advantage or gear ratio, so that the other follows as the inverse; throughout this text, we will define the mechanical advantage as the force advantage, in keeping with much of the biomechanical literature, $G = G_F = G_v^{-1}$.

Three properties of Equation (1) have catapulted *G* into arguably the dominant functional parameter in the study of skeletal functional morphology. First, it is mathematically simple. Second, it appears to permit comparative inference about musculoskeletal dynamics, a complex trait, through measurement of two anatomical traits that are often easily accessible. Third, despite this simplicity, the implied functional significance appears both straightforward and significant: *G* is the force amplification factor, and the inverse of the velocity amplification factor—Equations (1)–(2) encode a trade-off between amplifying force or velocity; “what is gained in force is lost in speed” (Galilei 2002). The magnitude of *G* may consequently be interpreted as an indication for how this trade-off has been resolved, that is, whether selection has acted to increase output force or speed. Because trade-offs can act both as constraints or drivers of adaptation (Arnold 1992; Herrel et al. 2009; Holzman et al. 2011; Garland et al. 2022; Burress and Muñoz 2023), *G* has played a key role in a rich body of literature on the macroevolutionary diversification of skeletal anatomy.

Early studies of skeletal anatomy considered the mechanical function encoded by *G* as an essential evolutionary driver of postural variations in ungulates (Gregory 1912); as determinant of limb function during locomotion (Gray 1944); and as primary explanation for the diversity of mammalian skeletal shape in general (Smith and Savage 1956). In the last three decades alone, *G* has been the principal functional metric in the study of the ecological speciation in Darwin’s finches (Herrel et al. 2009); the evolution of feeding mechanisms in fish (Westneat 1994, 2004); the evolution of skull form in bats (Santana et al. 2012; Dumont et al. 2014); the morphological diversification of jaw morphology in vertebrates (Anderson et al. 2011; Deakin et al. 2023) and of chela morphology in scorpions (Simone and van Der Meijden 2018); bite force estimation in extinct species (Sakamoto 2010; Sakamoto et al. 2019); the early evolution of biting–chewing performance in the hexapoda (Blanke 2019); the ecological and taxonomic diversification of crown mammals (Tseng et al. 2023), and the diet of Mesozoic mammals (Morales-García et al. 2021); and the variability of rates of phenoptypic evolution in cichlids (Burress and Muñoz 2023). There is probably no other biomechanical metric that has been measured as extensively, and that has served as foundation for such far-reaching comparative, ecological, and evolutionary conclusions.

### Is the force–velocity trade-off universal? A shift in perspective

The functional interpretation of *G* as arbiter of a force–velocity trade-off continues to dominate the literature, but it is neither the only interpretation nor is it universally accepted. Alternative but not mutually exclusive suggestions posit that the magnitude of *G* influences the efficiency of locomotion (Reilly et al. 2007; Ren et al. 2010; Usherwood 2013); that systematic variations in *G* with animal body size help ensure that static equilibrium can be maintained with equal muscle effort despite the systematic variation of the muscle-to-weight-force ratio (Biewener 1989a; Dick and Clemente 2017); or that an appropriate choice and dynamic variation of *G* can optimize muscle mechanical performance during running (Carrier et al. 1994, 1998), and maximize the mechanical energy than can be stored in elastic elements to enhance performance during explosive movements ( Roberts and Marsh 2003; Olberding et al. 2019). Dissenting interpretations, questioning whether *G* encodes a universal force–velocity trade-off, have existed since at least the late 1970s (Coombs 1978) and have grown both louder and more numerous in the last decade (McHenry 2010, 2012; McHenry and Summers 2011; Olberding et al. 2019; Osgood et al. 2021). What is the origin of the disagreement?

To understand the primary objection, note an essential subtlety in Equations (1)–(2): the force- and velocity advantage apply instantaneously, that is, at any specific point in time. Consider two musculoskeletal systems, identical except that one has twice the mechanical advantages of the other. The instantaneous interpretation of *G* implies that if the muscle in both systems exerts an instantaneous force $F_i$ and shortens with an instantaneous velocity *v*, then the instantaneous output force and velocity differ by factors of two as well—the system with the larger gear ratio exerts a larger net force, $F_o = 2 G F_i$, but moves with a smaller velocity, $u = v (2 G)^{-1}$ and vice versa; varying *G* trades instantaneous output force against instantaneous output velocity. This interpretation is certainly *instantaneously* correct (Arnold et al. 2011), and is not called into question (previous misunderstandings partially arose from the use of incorrect terminology; see below and McHenry 2010; Arnold et al. 2011; McHenry and Summers 2011; Osgood et al. 2021). But how did the muscle in both systems come to achieve the shortening velocity *v*? If the mass is initially at rest, then reaching *v* demands acceleration. Intriguingly, a larger mechanical advantage will increase the output force, $F_o = G F_i$, and with it, the acceleration, $a=F_o/m$, so that speed is gained more quickly and with less displacement. It is tempting, then, to ponder why a larger mechanical advantage should not make it easier to reach a larger velocity—the exact opposite of a force–velocity trade-off.

The usual counterpoint to this objection nicely mirrors the historical development of the understanding of “simple machines”: for Aristotle and Archimedes, to use a lever was to defy the laws of nature through the use of art, for a large weight could be made to move with minuscule force (Gatto 2017). Heron, in his *Mechanics*, was perhaps the first Western scholar to note that the decrease in required forces comes with a cost (Gatto 2017), but it was Galileo who firmly established this insight through the introduction of the concepts of power and work: instead of in “a certain manner deceiving nature,” gearing may reduce the force required to move an object, but it cannot alter the work a machine can do. It is this focus on muscle work, the integral of force with respect to displacement, that has called the universality of force–velocity trade-offs into question (McHenry 2010, 2012; McHenry and Summers 2011; Olberding et al. 2019; Osgood et al. 2021). Instead of asking “For a given instantaneous muscle force $F_i$, shortening speed *v*, and gear ratio *G*, what are the instantaneous output force $F_o$ and velocity *u*?”, as supposed by the instantaneous perspective, an energy perspective on gearing asks: “How does gearing influence the ability of muscle to do work, and how does it control the ‘transmission efficiency’ of muscle work into the kinetic energy?” (McHenry 2010, 2012; McHenry and Summers 2011; Olberding et al. 2019; Osgood et al. 2021). In other words, instantaneous quantities, such as force and velocity, are replaced with integral quantities, such as work or impulse; the focus lies no longer on a specific instant in time during a contraction, but on the contraction outcome. The key problem is that these two perspectives can be in conflict.

This conflict came to the fore in a series of papers published in the 2010s. In what was perhaps the first study to redirect the focus from an instantaneous interpretation of gearing to one that analyzed energy flow, McHenry (2010) argued that “there is no force–velocity trade-off in dynamic lever systems” and, consequently, that “skeletal geometry provides a necessary, but insufficient, means to characterize the dynamic performance of a lever system.” Arnold et al. (2011) countered, in no less certain terms, that “there is always a trade-off between speed and force in a lever system” and that the results of McHenry merely reflected the trivial conservation of energy. This critical counterargument was partially triggered by the use of “quasi-static” and “static equilibrium” by McHenry, although “instantaneous” was meant. McHenry and Summers (2011) clarified that the disagreement largely reflects one of emphasis and not of principle: force–velocity trade-offs exist instantaneously, as argued by Arnold et al. (2011), but they are not absolute, for they disappear when the work output of muscle is kept constant—instantaneous amplification does not necessarily map onto the outcome of a contraction (McHenry and Summers 2011). But Arnold et al. also questioned the assumption that muscle work input can be considered constant, and instead suggested that the mechanical metric of interest was muscle power, so that a force–velocity trade-off also persists dynamically (Arnold et al. 2011). McHenry then went one step further and demonstrated more formally what was already suggested in the discussion of the initial commentary: the velocity resulting from a contraction can vary with gear ratio *even when muscle supplies a constant amount of energy*, because gearing controls the transmission efficiency of the energy conversion, that is, how much of each unit of muscle work serves to increase kinetic energy (McHenry 2012; this observation is unpacked more formally below). Using forward dynamic simulations, McHenry showed elegantly that variations in gear ratio can leave the resulting speed unaffected, decrease it, or increase it, depending on the mechanical environment; there exists no one-to-one mapping between gear ratio and output speed, and variations in *G* are neither necessary nor sufficient to change the maximum speed a musculoskeletal system can impart (see also Coombs 1978; Nagano and Komura 2003; Lee et al. 2009; Richards 2011; Richards and Clemente 2013; Olberding et al. 2019; Osgood et al. 2021; Labonte 2023; Labonte et al. 2024, for related results).

Here, we take McHenry’s work as a starting point, and seek to develop a first-principles framework to analyze the influence of the mechanical advantage on the mechanical energy output and mechanical energy flow in musculoskeletal systems. Our analysis builds on the theory of physiological similarity (Labonte 2023), and differs from earlier work in two key aspects: First, instead of an “elastic actuator” akin to a spring, we will analyze the dynamics of a system driven directly by muscle. This difference will enable us to show that variations in mechanical advantage can control how much work muscle can deliver, so reconciling Arnold et al.’s objection with McHenry’s observation. Second, where others relied on numerical simulations (McHenry 2010, 2012; Olberding et al. 2019; Osgood et al. 2021), we will derive exact symbolic relationships wherever possible. By leaning on explicit analytical expressions rather than simulations, we will be able to derive results that readily generalize and sharpen our understanding of the physical principles at play. The aims of this work are to unambiguously pinpoint the role of gearing in modulating muscle energy output through a rigorous mechanical analysis; to investigate how gearing interacts with external forces to modulate the conversion of muscle work input into kinetic energy output; and to further clarify the conditions under which variations in *G* can be interpreted as an evolutionary adaptation that reflects force–velocity trade-offs across animal size and mechanical environment.

## Model formulation and assumptions

The aim of this manuscript is to analyze how gearing influences (i) the work muscle can deliver in a single contraction, and (ii) how it controls the distribution of each unit of muscle work among different forms of energy that characterize typical musculoskeletal systems—kinetic energy, heat, or gravitational potential energy may serve as three illustrative examples. A convenient physical approach for such an analysis is to invoke the conservation of energy, which relates the work *W* that was done to the change in energy $\Delta E$ that results:


(4) \begin{eqnarray*}
W = \Delta E.
\end{eqnarray*}

We use the conservation of energy to evaluate the flow of mechanical energy in the idealized model of a real musculoskeletal system that forms the basis of the theory of physiological similarity (Labonte 2023): a muscle with volume $V_m$ exerts a force $F_i(t)$, and acts on a mass *m* via an anatomical arrangement with characteristic constant mechanical advantage *G*; there are no in-series elastic elements (Fig. 1). The muscle can apply a force of no more than $F_{\text{max}}$, shorten by a distance of at most $\delta _{{\text{max}}}$, and no faster than with a maximum shortening velocity $v_{\text{max}} = L_m \dot{\varepsilon }_{\text{max}}$, where $L_m$ is a characteristic fascicle length, and $\dot{\varepsilon }_{\text{max}}$ is a maximum strain rate in units muscle lengths per second. Throughout the initial analysis, it is assumed that the muscle can exert its maximum force until it has contracted by $\delta _{\text{max}}$, or until it has achieved a shortening velocity $v_{\text{max}}$, $F_i(\delta \le \delta _{\text{max}}, v \le v_{\text{max}}) = F_{\text{max}}$; above this maximum shortening distance and shortening speed, the force drops instantaneously to zero, $F_i (\delta > \delta _{\text{max}}, v > v_{\text{max}}) = 0$. Implementing the force–length and force–velocity relationships as simple step functions is a simplification, and muscle does not behave in such an idealized way: the force it generates varies with both muscle length and muscle shortening speed before their limiting values are reached, $F_i (\delta > 0, v > 0) < F_{\text{max}}$. We will demonstrate at the end of the results section that such more complex relationships considerably increase mathematical complexity without changing the general physical picture, so retrospectively justifying the idealization.

To investigate how gearing can constrain the work output of muscle, we will first analyze the hypothetical case where muscle provides the only applied force in the system. Muscle then only does work against the inertial force, the product between mass and the instantaneous acceleration. Each unit of muscle work $W_m$ is thus entirely converted into kinetic energy, *K*:


(5) \begin{eqnarray*}
W_m = \Delta K.
\end{eqnarray*}

Although this scenario does not resemble any real system exactly, it is approximately correct as long as all external forces ($F_e$) are much smaller than the output force of the musculoskeletal system, $F_e \ll F_o$, which implies that $F_{\text{net}} = F_o - F_e \approx F_i G$, and thus $W \approx W_m$. More importantly, it will focus the attention on the mechanism through which gearing can curtail muscle work output, and so help to understand the results of more complex analyses downstream.

To investigate how gearing influences the conversion of muscle work into different types of energy, we will next increase system complexity, and allow external forces $F_e(t)$ to oppose the motion of the mass. Such forces are “parasitic” in the sense that they do negative internal work, $W_e$. The presence of external forces thus enforces a partitioning of each unit of muscle work into kinetic vs parasitic energy:


(6) \begin{eqnarray*}
W = W_m - W_e = \Delta K ,\\
W_m = \Delta K + \Delta E_e.
\end{eqnarray*}

Whereas the analysis of an idealized inertial contraction was used to explain how gearing can curtail muscle work output, this step will be used to build a conceptual understanding of how gearing can influence the distribution of muscle work across kinetic and parasitic energy.

The results from both analyses will then be combined to determine an estimate of the mechanical advantage that provides the best compromise between the two opposing demands they revealed: for muscle to deliver its maximum work capacity, the mechanical advantage cannot be arbitrarily large; but to avoid this delivery taking large amounts of time, and to maximize the increase in kinetic energy, *G* can also not be arbitrarily small.

In a last step, this result will be put to use to illustrate how “optimal” gearing depends on musculoskeletal anatomy, physiology, and the mechanical environment in which the contraction takes place. This demonstration will be conducted through the analysis of three simple case studies of real musculoskeletal systems.

## Results

### How can gearing influence muscle energy output?

For an inertial contraction starting from rest, conservation of energy implies


(7) \begin{eqnarray*}
W_m = K = \frac{1}{2} m u^2.
\end{eqnarray*}

The problem is then reduced to the question of how much energy muscle can deliver. This question is as old as biomechanics itself (Borelli 1680), and the canonical answer is that the maximum energy output of muscle, its work capacity $W_{\text{max}}$, is independent of the mechanical advantage. This assertion can be understood as follows: the work capacity of muscle depends on the maximum displacement-averaged force $\hat{F}_{i, \text{max}}$ it can exert as it shortens over a maximum contraction distance $\delta _{\text{max}}$, $W_{\text{max}} = \hat{F}_{i, \text{max}} \delta _{\text{max}}$. The work capacity of a geared muscle, in turn, is the product of the displacement-averaged external force $\hat{F}_{o,{\rm max}} = G\hat{F}_{i, {\rm max}}$ and the external displacement over which this force is moved, $x_{\rm max}= \delta _{\rm max}/G$. It follows that $\hat{F}_{o,max}x_{\rm max}= G\hat{F}_{i,max} \delta _{\rm max}/G = W_{\rm max}$; the work capacity of a geared muscle is the same as that of the ungeared muscle, and thus independent of the mechanical advantage.

If that was all there was to it, gearing would merely control how each unit of muscle work is split into the net displacement-averaged force, $\hat{F}_o = \hat{F}_i G$, vs the net displacement, $\delta = x G^{-1}$, but the muscle could deliver the same amount of work regardless of the value of *G*. Contractions would consequently always terminate at the same speed, the “Borelli-limit,” $u_{\text{Bo}} =\sqrt{2W_{\text{max}}/m}$ (Labonte 2023), and force–velocity trade-offs would only apply instantaneously but not ultimately (as suggested by McHenry 2010). But can the muscle deliver $W_{\text{max}}$ for any value of *G*?

To identify the problem with this assertion, note that the output speed *u* is directly coupled to the muscle shortening speed *v*, $u = v G^{-1}$. Inspect, then, the muscle shortening speed required to deliver the maximum work capacity for an arbitrary mechanical advantage, $v = G u_{\rm Bo} = G\sqrt{2W_{\text{max}}/m}$: as *G* diverges, so must the muscle shortening speed. But muscle cannot shorten with arbitrarily large speeds. Instead, muscle shortening velocity has an axiomatic upper limit, $v \le v_{\text{max}}$, so that the output speed is also bound by the “Hill-limit,” $u < u_{\text{Hi}} = v_{\text{max}} G^{-1}$ (Labonte 2023). This result is both elemental and crucial: muscle has not one but *two* characteristic energy capacities. The work capacity, $W_{\text{max}}$, is joined by a “kinetic energy capacity” (Labonte 2023; Labonte and Holt 2024),


(8) \begin{eqnarray*}
K_{\rm max}= \frac{1}{2} m u_{\text{Hi}}^2 = \frac{1}{2} m v^2_{\rm max}G^{-2}.
\end{eqnarray*}

**Fig. 2:**
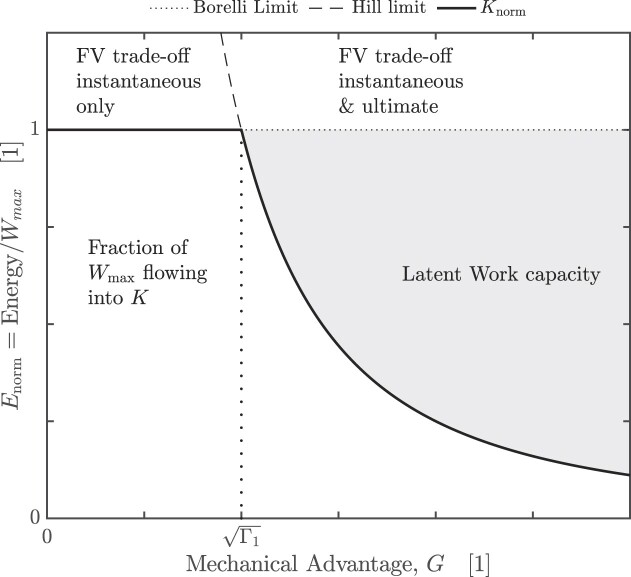
In an inertial contraction of an idealized musculoskeletal system, all muscle work is converted into kinetic energy, $W = W_m = K$. The mechanical energy landscape is split into two regimes, separated by the optimal mechanical advantage, $G_{\text{opt}} = \sqrt{\Gamma _1} = \sqrt{K_{\text{max}, 1}/W_{\text{max}}}$, with which the muscle can deliver maximum kinetic energy in minimal time. For any smaller mechanical advantage, $G \le G_{\text{opt}}$, the muscle can always deliver its maximum work capacity, defined by the Borelli-limit $W_m = W_{\rm max}$; force–velocity trade-offs thus only apply instantaneously, but do not map on the outcome of the contraction (McHenry 2010; Labonte 2023). In this regime, any reduction in *G* increases the time *t* required for the delivery of the same work $W_{\text{max}}$—as *G* becomes arbitrarily small, so does the maximum average muscle power, $P_{\text{max}} = W_{\text{max}} t^{-1}$, although the output velocity remains constant. For $G > G_{\text{opt}}$, in turn, the muscle contraction speed reaches $v_{\rm max}$ before the muscle has delivered $W_{\text{max}}$, so that muscle energy output is bound by the Hill-limit instead, $W_m = K_{\text{max}} < W_{\text{max}}$. A fraction $1-\Gamma$ of the muscle work capacity now remains “latent,” in the sense that the system can access it in principle, for example, if the payload is increased (which changes $\Gamma _1$), or indeed if *G* is decreased (which changes the kinetic energy capacity, $K_{\text{max}}$). Due to this latent work capacity, force–velocity trade-offs apply not only instantaneously, but also map onto the ultimate outcome of the contraction (Arnold et al. 2011; Labonte 2023). In this regime, a reduction in *G* thus releases work capacity, and this increase balances the increase in contraction time such that the maximum average power output of the muscle remains constant (Labonte 2023). The separation of the energy landscapes into the two regimes, delineated by the optimal mechanical advantage, reconciles the debate between McHenry, Arnold, and others (McHenry 2010, 2012; Arnold et al. 2011; McHenry and Summers 2011): both arguments are correct, but in opposite regimes of the physiological similarity index $\Gamma = \Gamma _1 G^{-2}$ (see text for details).

The work and the kinetic energy capacity pose two hard, independent bounds on muscle work output, $W_m \le W_{\text{max}}$ and $W_m \le K_{\text{max}}$, respectively. The maximum work muscle can deliver in a single contraction is consequently determined by whichever of the two limits is smaller (Labonte 2023 and Fig. 2). Crucially, and unlike $W_{{\rm max}}$, $K_{{\rm max}} \propto G^{-2}$ depends explicitly on the mechanical advantage, and can thus be geared. As long as $K_{\text{max}} < W_{\text{max}}$, gearing can therefore alter muscle energy output, and so affect the output speed that results from concentric muscle contractions (Labonte 2023; Labonte et al. 2024). In this regime, force–velocity trade-offs thus apply both instantaneously and ultimately, as argued by Arnold et al. (2011; Fig. 2).

It is convenient to determine an explicit relationship between mechanical advantage and muscle energy output in terms of the physical parameters of the musculoskeletal system. To find such a general condition, we first normalize Equation (7) by dividing both sides with $W_{\text{max}}$, which yields a dimensionless form of the conservation of energy:


(9) \begin{eqnarray*}
W_{\text{norm}} = K_{\text{norm}}.
\end{eqnarray*}

The crux is to recognize that both $W_{\text{norm}}$ and $K_{\text{norm}}$ are bound from above. The normalized work cannot exceed unity ($W_{\rm norm}\le 1$) because muscle has a limiting work capacity, and the normalized kinetic energy cannot exceed $K_{\text{norm}} \le K_{\text{max}} W_{\text{max}}^{-1}$, because muscle has a limiting kinetic energy capacity. The ratio of the two characteristic energy capacities, $\Gamma = K_{\rm max}W_{\rm max}^{-1}= \frac{1}{2}m v_{\rm max}^2 G^{-2} W_{\rm max}^{-1}$, is the dimensionless “physiological similarity index,” so called because idealized musculoskeletal systems that operate with equal $\Gamma$ put out equal fractions of their maximal displacement, speed, work, and power capacity in a single contraction (Labonte 2023).

Inspection of $\Gamma$ permits direct identification of the dominant physical constraint on muscle energy output: if *G* is very large, then $\Gamma < 1$, and thus $K_{\text{max}} < W_{\text{max}}$. The muscle is blocked from delivering its full work capacity, $W_{\rm norm}= \Gamma < 1$, because it does not have a sufficiently high kinetic energy capacity; force–velocity trade-offs apply both instantaneously and ultimately (Fig. 2). If *G* is very small, in turn, $\Gamma > 1$, and thus $K_{\text{max}} > W_{\text{max}}$—the muscle can deliver its full work capacity, $W_{\rm norm} = 1 < \Gamma$, so that force–velocity trade-offs apply instantaneously, but not ultimately. The introduction of the physiological similarity index therefore reconciles the opposing views of McHenry and Arnold et al. (Arnold et al. 2011; McHenry and Summers 2011)—both are correct, but in two opposite “design” regimes, characterized by small or large values of $\Gamma$, respectively. For an idealized muscle in an inertial contraction, the transition between both regimes occurs at $\Gamma = 1$, that is, at the unique gear ratio for which the work and kinetic energy capacity of the musculoskeletal system are equal (Labonte 2023):


(10) \begin{eqnarray*}
G (\Gamma =1) = \sqrt{\frac{m v^2_{\text{max}}}{2 W_{\text{max}}}} = \sqrt{\Gamma _1},
\end{eqnarray*}

where $\Gamma _1 := \Gamma (G=1)$ is the physiological similarity index for a muscle with a mechanical advantage of unity.

The key result of this first analysis may be summarized as follows: for muscle to deliver maximum energy, its kinetic energy capacity must be at least as large as its work capacity, $\Gamma \ge 1$. Because the kinetic energy capacity can be geared, this condition can be achieved for any musculoskeletal system through appropriate gearing, $G \le \sqrt{\Gamma _1}$ (Fig. 2).

### How does gearing influence energy flow?

Consider next the more general case where muscle contracts against non-negligible external forces, $F_e$, which oppose motion. These external forces do negative work, and so redirect muscle work from kinetic into other forms of energy, $E_e$ (Scholz et al. 2006; McHenry 2012; Labonte 2023). It is not immediately obvious how a fixed amount of muscle work will be split between *K* and $E_e.$ To determine this split, we re-write the energy balance [Equation (6)] to find:


(11) \begin{eqnarray*}
K = W_m(1 - W_e/W_m) = W_m(1- \hat{\kappa }).
\end{eqnarray*}

The key difference to the inertial case governed by Equation (7) is the dimensionless quantity $\hat{\kappa }$, referred to as the “reduced parasitic energy” in the framework of physiological similarity (Labonte 2023). Without loss of generality, $\hat{\kappa }$ is the fraction of muscle work flowing into work against external forces. The “hat” symbol reflects that this fraction is also equal to the ratio between the displacement-averaged opposing and driving forces, respectively,


(12) \begin{eqnarray*}
\hat{\kappa } = \frac{W_e}{W_m} = \frac{\int F_e d x}{\int F_o d x} = \frac{\hat{F}_e}{\hat{F}_i G}.
\end{eqnarray*}

In the presence of external forces, it thus becomes important whether a unit of muscle work is delivered by displacing a large external force over a small displacement, or by moving a small external force by a large displacement. The fraction of each unit of muscle work that flows into kinetic energy is maximized if it is delivered with maximal displacement-averaged driving force, $\hat{F}_o = \hat{F}_i G$, and vanishing displacement $x = \delta /G$, that is, for a diverging mechanical advantage.

**Fig. 3:**
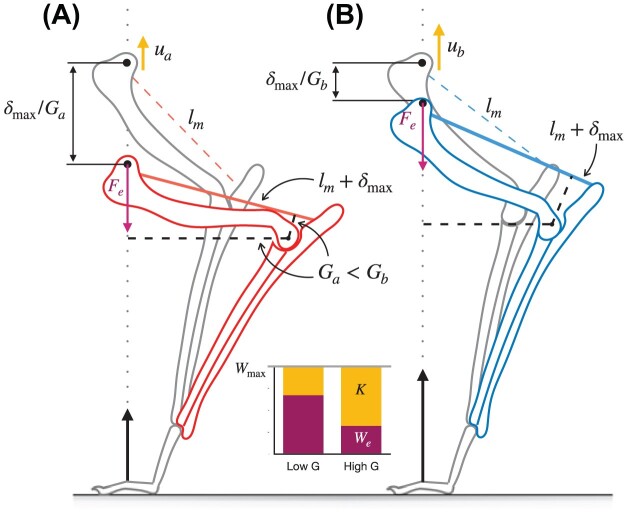
A thought experiment with two jumping animals illustrates how the magnitude of the mechanical advantage, *G*, controls the partitioning of muscle work into kinetic energy, *K*, vs parasitic work, $W_e$; the morphology is based loosely on the mammalian forelimb. The two animals (**A**) and (**B**) are identical, except that (**A**) has a lower mechanical advantage at the elbow, $G_a < G_b$. Let the muscles of both (**A**) and (**B**) deliver their maximum work capacity $W_{\rm max}$: the elbow extensors are first maximally stretched to a length $l_m+\delta _{\rm max}$ (in color), and then contract to accelerate the center-of-mass until both animals have returned to their initial posture (in gray). Because $G_a < G_b$, animal (**A**) must crouch lower to stretch and contract its muscles by the same amount, so that the gravitational force resists movement over a larger external distance $\delta _{\text{max}}/G_a$ during the acceleration phase. As a consequence, the work demanded by the necessary change in gravitational potential energy during the acceleration is larger in (**A**), $W_e = m g \delta _{\text{max}}/G_a$, so that less work is available to increase the kinetic energy $K = W_{\text{max}} - W_e$ (chart inset). Consequently, animal (**B**) achieves a higher takeoff speed $u_b$ at the same takeoff height, and thus jumps higher above standing: a higher mechanical advantage results in a larger average net force, *and* a larger take-off velocity, in defiance of a force–velocity trade-off (see also Scholz et al. 2006; McHenry 2012; Labonte 2023; Labonte et al. 2024). This particular effect of *G* may be generalized for any external force through the introduction of the reduced parasitic energy $\hat{\kappa }$, a dimensionless number that defines the transmission efficiency of muscle work into kinetic energy, $\eta = K/W_m = 1 - \hat{\kappa }$ (see text for further details).

To gain a more intuitive physical understanding of this mathematical result, consider a concrete example of two animals that seek to jump off the ground with a maximal takeoff velocity *u* (Fig. 3). All else being equal, let one animal exhibit a smaller and the other a larger mechanical advantage. The muscles in both cases contract by the same amount, so that the work they deliver is identical, and equal to $W_{\rm max}$. However, the external movement that results from an equal amount of muscle shortening differs; it is larger for the animal with the smaller mechanical advantage. The work done against gravity is the product of this distance and the gravitational force, and is consequently larger, too. Because the total muscle work is identical across both cases, a difference in gravitational potential energy costs $W_g$ must imply a difference in the change in kinetic energy, $\Delta K = W_m - W_g$. Thus, the larger mechanical advantage results in both a larger average force and a larger take-off velocity, in defiance of the force–velocity trade-off; the height gained after take-off is higher for the jump that involved a higher mechanical advantage.

The key result of this second step of the analysis may then be summarized as follows: movement is generally opposed by external forces, and these forces demand a share of muscle work; the work done by these external forces is equal to the product between their displacement-average, $\hat{F}_e$, and the external displacement, *x*; and the mechanical advantage dictates the magnitude of the external displacement per unit muscle work. As a result, *G* controls the partitioning of muscle work into kinetic vs “parasitic” forms of energy, that is, the transmission efficiency, $\eta = K/W_m = 1 - \hat{\kappa }$(McHenry 2012).

### What constitutes optimal gearing?

It was demonstrated that gearing controls both the fraction of $W_{\text{max}}$ that muscle can deliver, and how each unit of delivered work is partitioned into different forms of energy. For muscle to deliver $W_{\text{max}}$, the mechanical advantage cannot be arbitrarily large—it must be small enough to ensure that $W_{\text{max}}$ has been delivered exactly when or before $v_{\text{max}}$ is reached. Although any mechanical advantage smaller than this critical condition will allow muscle to deliver $W_{\text{max}}$, reducing *G* any further would bring two main disadvantages. First, it would reduce the average output force, so that delivering $W_{\text{max}}$ will take longer—a musculoskeletal system operating exactly at the critical *G* delivers maximum work in minimal time, and thus maximizes work and average power output simultaneously (Labonte 2023). Second, decreasing *G* also results in an increased loss of muscle work to parasitic forces, that is, it reduces the transmission efficiency from work into kinetic energy, and consequently the output speed muscle can impart. There thus should exist an intermediate mechanical advantage that represents the optimal resolution of these competing demands (McHenry 2012; Olberding et al. 2019; Labonte 2023).

To find this “optimal” mechanical advantage, we first obtain a dimensionless form of Equation (11). Division with $W_{\text{max}}$ yields


(13) \begin{eqnarray*}
W_{\text{norm}} = \frac{K_{\rm norm}}{1- \hat{\kappa }}.
\end{eqnarray*}

**Fig. 4:**
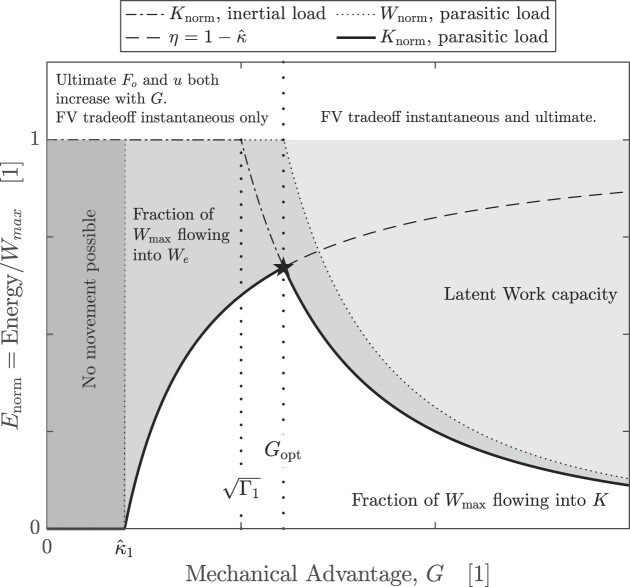
The energy landscape for an idealized musculoskeletal system in a contraction against a constant external force $F_e$. The external force consumes a fraction $\hat{\kappa }= \hat{\kappa }_1/G$ of the muscle work, and so alters the appearance of the energy landscape in two main ways. If the mechanical advantage drops below a critical value $G_c < \hat{\kappa }_1$, positive acceleration is no longer possible, as the muscle cannot overcome the external force. For $G > G_c$, *G* controls the fraction of the muscle work that flows into kinetic energy, $\eta = K/W_m = 1-\hat{\kappa }_1/G$, or the “transmission efficiency” of the musculoskeletal system (McHenry 2012; Labonte 2023). The optimal mechanical advantage $G_{\rm opt}$ [Equation (15)] now not only allows the system to deliver maximum work in minimal time, but is also the unique gear ratio for which the increase in *K* is maximized. Consequently, $G_{\rm opt}$ depends explicitly not only on $\Gamma _1$, as for an inertial contraction, but also on $\hat{\kappa }_1$, the ratio of the displacement-averaged opposing and driving force [see Equation (15)]. For $G > G_{\rm opt}$, the muscle work output is limited by its kinetic energy capacity. Note that this specific energy landscape is for $\Gamma _1 = 1$ and $\hat{\kappa }_1 = 0.4$; the landscape appearance depends on the relative magnitude of $\Gamma _1$ and $\hat{\kappa }_1$, and is subtly different for parasitic forces that vary with speed, because equilibrium of forces now only occurs dynamically (see Fig. 5F for an example).

The optimal mechanical advantage of an idealized muscle is the critical value of *G* for which the maximum work capacity is delivered ($W_{{\rm norm}} = 1$) exactly when the maximum shortening speed is reached $(K_{{\rm norm}} = \frac{K_{\rm max}}{W_{\rm max}} = \Gamma$; Fig. 4). Equation (13) can thus be written as


(14) \begin{eqnarray*}
1 = \frac{\Gamma }{\left(1 - \hat{\kappa }\right)} = \frac{\Gamma _1}{G_{\rm opt}^2(1-\hat{\kappa }_1/G_{\rm opt})},
\end{eqnarray*}

where we define $\hat{\kappa }_1 := \hat{\kappa }(G=1)$ as the reduced parasitic energy of a musculoskeletal system with a gear ratio of unity (thus $\hat{\kappa }_1/G = \hat{\kappa }$), and note that $\Gamma = \Gamma _1/G^2$. Equation (14) is quadratic in $G_{\rm opt}$, and the positive root reads


(15) \begin{eqnarray*}
G_{\text{opt}} = \frac{1}{2} \hat{\kappa }_1 \left(1 + \sqrt{4 \frac{\Gamma _1}{\hat{\kappa }_1^2} + 1}\right),
\end{eqnarray*}

Note that for all but the case of a constant external force, this expression is implicit, as $\hat{\kappa }_1$ depends on *G* (see below for a more detailed discussion of this point).

The ratio $\Gamma _1/\hat{\kappa }_1^2$ emerges as a key determinant of the optimal mechanical advantage. Two limiting cases may be considered. When $\Gamma _1/\hat{\kappa }_1^2 \rightarrow 0$, external forces are large relative to the system inertia; and when $\Gamma _1/\hat{\kappa }_1^2 \rightarrow \infty$, external forces are negligible. The optimal mechanical advantage in each limit follows as


(16) \begin{eqnarray*}
\lim _{\Gamma _1/\hat{\kappa }_1^2 \rightarrow 0 } G_{\rm opt} &= \hat{\kappa }_1,
\end{eqnarray*}

(17) \begin{eqnarray*}
\lim _{\Gamma _1/\hat{\kappa }_1^2 \rightarrow \infty } G_{\rm opt} &= \sqrt{\Gamma _1}.
\end{eqnarray*}

Both results may be interpreted as an equilibrium between the two dominant displacement-averaged forces (see also Richards and Clemente 2013; Labonte 2023). When the external force is dominant, *G* is optimal when $\hat{F}_o = \hat{F}_e$ (for $\Gamma _1/\hat{\kappa }_1^2 \rightarrow 0$)—and indeed, no motion is possible, if ever $\hat{F}_o < \hat{F}_e$. When inertial forces dominate, $\hat{F}_o = \hat{F}_{\rm {inertial}} = K_{\rm max} \delta _{{\rm {max}}}^{-1}G^{-2}$ (for $\Gamma _1/\hat{\kappa }_1^2 \rightarrow \infty$). In many realistic scenarios, $G_{\text{opt}}$ will fall between these two limits, and is defined exactly by Equation (15).

The key result of this third step of the analysis may then be summarized as follows: *G* should neither be too large, for otherwise muscle can only deliver a fraction of its work capacity, nor can it be too small, for otherwise the time it takes to deliver each unit of work diverges, and most of it is lost to parasitic energy (Fig. 4). These competing demands are encoded in two dimensionless numbers, which, in combination, define the optimal mechanical advantage: the value of *G* with which muscle can deliver maximum work in minimal time, and which results in the maximal possible increase in the system’s kinetic energy.

Up to now, the analysis was agnostic to the nature of the external forces, and thus general; the aim was to build intuition for the key physical mechanisms at play, and to develop a theoretical framework that reveals the effect of gearing on the flow of mechanical energy within the system under study. We next apply this framework to three case studies to both illustrate its use and probe its explanatory power: the strike of a praying mantis, as an example for an approximately inertial contraction; the jump of kangaroo rat, as an example for a contraction against the constant gravitational force; and the leg stroke of a frog, as an example for a contraction against a velocity-dependent external force. We remind the reader of the simplifying assumption that the muscle force remains constant at its maximum throughout the contraction; this idealization is discussed briefly and critically at the end.

### Case studies

**Fig. 5:**
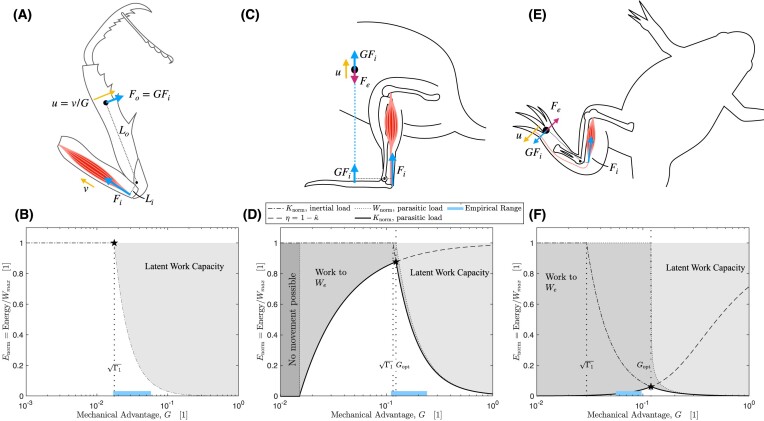
Three case studies illustrate how the mechanical environment influences the energy landscape for an idealized musculoskeletal system. In all cases, the optimal mechanical advantage, $G_{\rm opt}$, is defined as the value of *G* that enables the musculoskeletal system to deliver maximum work in minimal time, and with maximal kinetic energy change (black stars). (**A**) The speed of a praying mantis strike is limited by the work muscle can do against an inertial load; external forces are negligible. It follows that $G_{\rm opt} = \sqrt{\Gamma _1}$ (black star). (**B**) A direct prediction falls within the range of empirical estimates for the mechanical advantage of the major coxo-femoral extensors in the mantis forelimb (bar along horizontal axis, in color; see Supplementary Information for details). (**C**) The takeoff jump speed of a kangaroo rat is determined by the work muscle can do against the inertial force and the gravitational force. (**D**) $G_{\rm opt}$ now depends on two dimensionless numbers, as defined by Equation (15). A direct prediction (star) falls within the range of empirical estimates (horizontal bar; see Supplementary Information for details). (**E**) A frog swimming in a fluid medium contracts the plantaris muscle to push its foot rapidly through the water. (**F**) The empirical estimate for the mechanical advantage (horizontal bar) is now far larger than the inertial optimum $\sqrt{\Gamma _1}$, and close to the directly predicted optimum $G_{\rm opt}$ (black star; see Supplementary Information for details). The energy landscape here differs from the gravitational case (panel D), as the external forces are velocity-dependent. As a result, there exists no minimal *G* below which movement is impossible; equilibrium of forces only occurs dynamically. Furthermore, $W_e$ is not “wasted” *per se*, but a portion is transmitted to the body as kinetic energy. Due to the velocity-dependence of the external force, the mechanical advantage that maximizes foot kinetic energy also maximizes average and peak output force.

Praying mantises exhibit a conspicuous adaptation of their forelimbs into a raptorial appendage, capable of catching prey in a predatory strike lasting <100 ms (Rossoni and Niven 2020). To catch prey effectively, they must be able to achieve maximum speed in minimal time—how should the raptorial appendage be geared to achieve this? Given the vertical posture of the forelimbs (Fig. 5A) and small appendage mass (0.17 g for an adult *Heirodula membranacea*; Gray and Mill 1983), the external forces are likely small compared to the muscle force, and so $\hat{\kappa }\approx 0$. The strike occurs with $\Gamma _1 \approx 3.1\times 10^{-4}$ (see Supplementary Information), and the optimal gear ratio follows as $G_{\text{opt}} = \sqrt{\Gamma _1} \approx 0.018$, within the range observed during the movement $G_{\text{emp}} \approx [0.017,0.058]$ (Fig. 5B; Gray and Mill 1983).

With a body mass of 106 g, the desert kangaroo rat is considerably heavier than the preying mantis forelimb. Instead of being a dangerous predator, its hindlimbs perform quick escape jumps, which may serve as an example for a contraction against a non-negligible gravitational force, $F_e = m g$ (Fig. 5C). Assuming that jumps are driven dominantly with the ankle extensors, we estimate $\Gamma _1 \approx 0.013$ (using data from Rankin et al. 2018; Javidi et al. 2020; see Supplementary Information)—about 30 times greater than for the mantis raptorial appendage. The external force is constant, so that the reduced parasitic energy follows as $\hat{\kappa }_1 = m g /F_i \approx 0.015$. Equation (15) provides an explicit condition for the optimal mechanical advantage, the value of *G* which maximizes the take-off velocity, which yields $G_{\text{opt}} \approx 0.12$; again within the range of empirical estimates, $G_{\text{emp}} \approx [0.11,0.24]$ (Fig. 5D) based on anatomical moment arms about the ankle (Biewener and Blickhan 1988; Rankin et al. 2018; see Supplementary Information). Note that although the kangaroo rat is considerably heavier than the praying mantis forelimb, gravity still only plays a relatively minor role in the energy landscape, and the optimal mechanical advantage remains close to the inertial limit $G_{\rm opt} = \sqrt{\Gamma _1} \approx 0.11$.

Consider last the frog *Xenopus laevis*, which uses drag-based propulsion from its hind legs to swim through water (Fig. 5E). We seek to estimate the mechanical advantage of the plantaris that enables maximum foot speed. The contraction occurs with $\Gamma _1 \approx 9 \times 10^{-4}$ (based on data from Richards 2008; Richards and Sawicki 2012; Clemente and Richards 2013; see Supplementary Information), and, because the movement takes place in water, gravity can be neglected, but a drag force $F_D$ must be considered. For *Xenopus* foot strokes, the drag force varies approximately with the square of the speed, $F_D = \beta _Q u^2$ ($\beta _Q$ is a constant quadratic drag multiplier of dimension kg m$^{-1}$; Richards and Sawicki 2012). Thus $\hat{\kappa }= \hat{F}_D (\hat{F}_i G)^{-1}$. However, because the displacement-averaged external force now depends on speed ($\hat{F}_e = \hat{F}_D(u)$), it also depends on *G*, so that Equation (15) is only implicit.

To the best of our judgment, no explicit and symbolic expression for $G_{\text{opt}}$ exists for contractions against a quadratic drag force (see Supplementary Information). However, the consideration of two limits is instructive. If the drag force is small compared to the inertial force, the optimal mechanical advantage approaches $\sqrt{\Gamma _1}$ [Equation (17)]; the system behaves approximately as if it was purely inertial. If the drag force is large compared to the inertial force, *G* is optimal if the muscle reaches its maximum shortening velocity exactly when the drag force and muscle output force $F_o = F_{{\rm max}} G$ are equal (see Supplementary Information). For a quadratic drag force, it follows


(18) \begin{eqnarray*}
\lim _{\kappa _{Q\text{max}, 1}/\Gamma _1 \rightarrow \infty } G_{\text{opt}} = \left({\frac{\beta _Q v^2_{\rm max}}{F_{\rm max}}}\right)^{1/3} = \sqrt[3]{\kappa _{\text{Qmax}, 1}},
\end{eqnarray*}

where $\kappa _{Q\text{max}, 1}$ is the ratio between the maximum ungeared drag and driving force, respectively. For the frog limb stroke, we estimate $\kappa _{Q\text{max},1} \approx 1.7\times 10^{-3}$ (based on a drag coefficient of 2; Richards and Clemente 2013), so that $G_{\rm opt} = 0.03$ in the inertial limit and $G_{\rm opt} \approx 0.12$ in the force limit. An empirical estimate of $G_{\rm emp} \in [0.06,0.1]$ is extracted from the range of moment arm values provided in Clemente and Richards (2013) and Richards and Clemente (2013); see Supplementary Information. The estimate lies between the two limits, but is closer to the force limit, for which we predict a maximum foot velocity ($v_{\rm max}/G_{\rm opt}$) of 1.2 m s^−1^, close to the empirically measured speed of 1 m s^−1^ (Richards 2010). An explicit symbolic expression for the optimal gear ratio can be found for a linear drag force, which can then be used to approximate quadratic drag by equating the upper $G_{\rm {opt}}$ limits. This approximation is shown in Fig. 5F. Note that the predicted $G_{\rm opt}$ does not differ from Equation (18), because the system is in dynamic equilibrium.

It may seem at first seem surprising that the frog leg “loses” most of its muscle work to the surrounding fluid, although its mechanical advantage is very close to the estimated optimum. However, this work is not lost proper, but is instead used to transfer kinetic energy to the body’s center of mass; the drag force for the frog’s foot during swimming is functionally similar to the ground reaction force during running. The center-of-mass transmission efficiency of the frog swimming stroke depends on the foot design, body drag force, body mass, and the kinematics of the motion (Richards and Clemente 2013), and is more complex to evaluate.

No mathematical complexity should distract from the generality of the key result illustrated with these case studies: the mechanical advantage controls the fraction of the maximum work capacity muscle can deliver, because it determines the relative importance of the work and kinetic energy capacity; and it defines the fraction of work that flows into kinetic energy, because it controls how work is partitioned between force and displacement. These two constraints are encoded by two dimensionless numbers: the ungeared physiological similarity index, $\Gamma _1$, which depends explicitly on muscle physiology and musculoskeletal anatomy; and the ungeared reduced parasitic energy, $\hat{\kappa }_1$, which reflects the influence of external forces and thus the physical characteristics of the specific environment in which the contractions take place. The two dimensionless numbers can be combined to predict the magnitude of the optimal mechanical advantage for real musculoskeletal systems, and, in three case studies, these predictions were either within or close to the range of empirical estimates.

### Further complexity: the force–velocity relationship of striated muscle

We have thus far analyzed the flow of mechanical energy for a muscle with a force–velocity relationship idealized as a step function: the muscle exerts its maximum force until its maximum shortening speed is exceeded, $F_i(v \le v_{\text{max}}) = F_{\text{max}}$. A more realistic assumption is that the muscle force is maximal only for an isometric contraction $F_i(v = 0) = F_{\text{max}}$, and drops in some fashion with speed until it hits zero at the maximum contraction speed, $F_i (v = v_{\text{max}})= 0$. How would consideration of such Hill-type muscle properties alter the key conclusions of this work?

The main investigative tool of this work has been the conservation of energy, $K = W_m(1- \hat{\kappa })$. This conservation principle remains valid no matter the exact relationship between muscle force and velocity; it is completely general. As a consequence, the trade-offs that result in an optimal *G* must remain in place: if *G* is very large, muscle can only deliver a vanishing fraction of its maximum work capacity, and if *G* is very small, delivering any work takes an excessive amount of time, and the transmission efficiency goes to zero. However, the physical definition of $G_{\text{opt}}$ is now more subtle, and determining its magnitude mathematically is significantly harder.

To understand the difficulty, note that the limiting values $F_i(v = 0) = F_{\text{max}}$ and $F_i (v = v_{\text{max}})= 0$ can only be reached in the limit of an infinitely slow (quasi-static) or infinitely fast (quasi-instantaneous) contraction (i.e., for $\Gamma \rightarrow \infty$ and $\Gamma \rightarrow 0$, respectively; Labonte 2023). As a result, the optimal mechanical advantage can no longer be defined as the value of *G* for which $K_{\text{max}}$ and $W_{\text{max}}$ are delivered simultaneously—no such contraction can exist. Instead, a conceptually equivalent $G_{\text{opt}}$ may be defined as the mechanical advantage at which muscle delivers the most work in the least time (note well that this is distinct from requesting maximum time-averaged power, which is necessary, but not sufficient for *G* to be optimal; Labonte 2023); to the best of our judgment, finding this optimal mechanical advantage likely requires numerical evaluation even for the most simple case of a linear force–velocity relationship (Labonte 2023).

Because $W_{\text{max}}$ and $K_{\text{max}}$ now only place an asymptotic upper bound on the muscle energy output, the energy landscapes remain qualitatively, though not quantitatively identical to the idealised case (see Supplementary Information for examples). The subtleties of the quantitative differences merit future investigation. However, the optimal *G* for delivering maximum kinetic energy still represents a trade-off between how much work muscle can deliver, how long it takes to do so, and how much of it flows to parasitic energy (Supplementary Fig. S1); the principal physical results presented above remain intact .

## Discussion

Movement is integral to animal behavior, can make the difference between life and death, and may demand the majority of an animal’s energy expenditure (Dickinson et al. 2000; Wilson et al. 2013, 2018). It therefore stands to reason that animal physiology and anatomy should bear the marks of selection for improved locomotor performance and efficiency. The mechanical advantage of musculoskeletal systems modulates the effect of muscle action on limb movement, and is thus one “design” element upon which selection may act. But what is the right mechanical advantage to select for?

An instantaneous analysis points to the force–velocity trade-off inherent in lever mechanics, and provides the basis for the most common functional interpretation of *G*—systems specialized for force should have large $G,$ and systems specialized for speed should have small *G*. But recent work has demonstrated elegantly and unambiguously that instantaneous transmission does not necessarily map onto the ultimate outcome of a muscle contraction (McHenry 2010, 2012; Osgood et al. 2021). Inspired by this “energy perspective” on gearing, and aided by the theory of physiological similarity for muscle-driven motion (Labonte 2023), we have conducted a general mechanical analysis of the energy flow in a minimalist musculoskeletal system. This analysis yielded two key results. First, although gearing leaves the maximum work capacity of muscle unaffected, it can prevent muscle from delivering it, because it modulates the muscle’s kinetic energy capacity. Second, gearing also controls the partitioning of each unit of muscle work into kinetic vs parasitic energy, that is, it influences the musculoskeletal transmission efficiency. For muscle to deliver $W_{\text{max}}$, *G* must be sufficiently small, but for this work to be delivered rapidly, and to ensure that most of it flows into kinetic energy, *G* must be sufficiently large. The relationship between the mechanical advantage and the achievable output speed is thus not a simple proportionality, as implied by the instantaneous perspective, but a complex function that depends on musculoskeletal anatomy, physiology, and the physical environment in which the contraction takes place (McHenry 2010, 2012).

Where the instantaneous perspective enables a functional interpretation of the mechanical advantage only through direct comparison—system *A* has a lower *G* than system *B*, and has therefore been selected for speed—the theory of physiological similarity permits a direct estimation of the specific mechanical advantage that allows musculoskeletal systems to deliver $W_{\text{max}}$ in minimal time, and with maximal output speed. In musculoskeletal systems that have been selected for dynamic movements, this mechanical advantage may reasonably be considered optimal (Olberding et al. 2019; Labonte 2023). In the following discussion, we will put this optimality criterion to work to analyze three different aspects of the functional anatomy of musculoskeletal systems: why the mechanical advantage is usually smaller than unity, why it may vary systematically with size, and the conditions under which it may be said to encode a force–velocity trade-off.

### Borelli’s riddle, and the magnitude of the optimal mechanical advantage

*Who indeed would be stupid enough to [...] use a machine or contrivance not to save forces but rather to spend forces? -*Giovanni Alfonso Borelli (1680)

Functional interpretations of *G* are typically grounded in an instantaneous perspective: specialization for speed requires small *G*, and specialization for force requires large *G*. Irrespective of whether this interpretation is sound, it has two shortcomings: it reveals nothing about the actual magnitude of a functionally optimized mechanical advantage; and, consequently, it can only ever allow functional interpretation through comparison. It was first observed by Borelli (1680) and has been confirmed many times since that the mechanical advantage of most musculoskeletal systems is smaller than unity, that is, gearing usually amplifies displacement and velocity, and thus attenuates force. But why should evolution be “stupid enough” to select systems with small mechanical advantages (Borelli 1680; Pope 2005)?

To answer “Borelli’s riddle,” we posit that selection should often favor mechanical advantages that enable muscle to deliver its maximum work capacity in minimal time, and that ensure maximal output speeds. In such a system, *G* can neither be too large, for muscle would then only have access to a fraction of $W_{\text{max}}$, nor can it be too small, for otherwise it would take longer to deliver $W_{\text{max}}$, and an increasing amount of muscle work would flow into parasitic instead of kinetic energy (Labonte 2023). The “optimal” mechanical advantage should thus be of intermediate magnitude. We have shown that this magnitude can be expressed in terms of two characteristic dimensionless numbers: the physiological similarity index, $\Gamma _1$, and the reduced parasitic energy, $\hat{\kappa }_1$ [Equation (15)]. Because these numbers are formed from the physiological and anatomical properties of the musculoskeletal system, the payload, and the physical environment in which the contraction takes place, there exist complex interdependencies, and the magnitude of *G* alone says little about mechanical performance.

To investigate what a focus on mechanical energy reveals about Borelli’s riddle, we will estimate the optimal gear ratio for the simplest case: a constant-force muscle contraction against an inertial load, that is, all external forces are of negligible magnitude. Although this may sound artificial, it is likely applicable to a large range of musculoskeletal systems, for the muscle force is much larger than the weight force in all but the heaviest vertebrate animals (Alexander 1985; Labonte 2023; Labonte et al. 2024).

In an inertial contraction, a muscle will deliver maximum work in minimal time if the ratio between its kinetic energy and work capacity is unity (Labonte 2023). For the minimalist musculoskeletal system studied in this work, this condition can be written as


(19) \begin{eqnarray*}
G_{\text{opt}}^2 = \left(\frac{L_i}{L_o}\right)^2 = \frac{E_{\text{max}}}{W_{\text{max}}} = \left[\frac{1}{L_m \varepsilon _{\text{max}}}\right] \left[\frac{m v_{\text{max}}^2}{2 F_{\text{max}}}\right],
\end{eqnarray*}

where $\varepsilon _{\text{max}}$ is the maximum muscle strain. The right-hand side was written as the product of two fractions to illustrate that the mechanical advantage is optimal if the squared ratio of the two anatomical lengths that define it is equal to the ratio between two physiological lengths: the maximum muscle shortening distance, $L_m \varepsilon _{\rm max}$, and the distance over which an ungeared muscle would have to shorten to accelerate to its maximum shortening speed, $m v^2 (2 F)^{-1}$. The mechanical advantage effectively acts as a modulator of this second term, such that an appropriate choice of *G* ensures that the two lengths are equal at the system level. In order to provide a rough estimate of $G_{\rm opt}$, we find that for a representative striated muscle (see Supplementary Information)


(20) \begin{eqnarray*}
G_{\text{opt}} \approx \frac{1}{4} L_m \rm \space{m}^{-1}.
\end{eqnarray*}

Thus, the characteristic muscle fascicle length $L_m$ would have to exceed 4 m to demand $G_{\rm opt} \,\gt\, 1$, providing a speculative answer to Borelli’s riddle, grounded in first principles: the mechanical advantage of most musculoskeletal systems “spends forces,” that is, is smaller than unity, because larger gear ratios would constrain muscle work output to be sub-maximal.

Because *G* is a geometric parameter that can help to navigate physiological constraints, we suggest to interpret it as an anatomical adaptation that relaxes selective pressure on muscle anatomy: through appropriate gearing, optimal performance can be achieved for any value of $L_m$. For a fascicle length of 10 cm, $G_{\text{opt}} \approx 0.03$, and for $L_m = 1$ cm, it drops to $G_{\rm opt} \approx 0.003$—although the fascicle length differs by a factor of 10, both systems can achieve equivalent mechanical performance, provided that the magnitude of $\Gamma$ is identical, so that they are physiologically similar (Labonte 2023); gearing provides an architectural degree of freedom in musculoskeletal “design.” The strong size-dependence of $G_{\rm opt}$ is apparent, and the focus of the next section.

### Osborn’s conjecture, and the size-dependence of the optimal mechanical advantage

*The straightening of the limb [in large quadrupeds] is an adaptation designed to transmit the increasing weight through a vertical shaft. -*Henry Fairfield Osborn (1900)

The optimal mechanical advantage for an idealized muscle is determined by the interplay of two dimensionless numbers: the ungeared physiological similarity index, $\Gamma _1$, defined by the physiological and anatomical properties of the muscle and the payload; and the ungeared reduced parasitic energy, $\hat{\kappa }_1$, which depends also on the specific physical environment in which the contraction takes place. How do both numbers vary with animal size?

Under the parsimonious assumptions of isogeometry and isophysiology, $\Gamma _1 \propto m^{2/3}$ (Labonte 2023). The scaling of $\hat{\kappa }_1$ in turn depends on the nature of the external force. For the gravitational force, of relevance for terrestrial locomotion, isogeometry implies $\hat{\kappa }_1 \propto m^{1/3}$; for a linear drag force one may find $\hat{\kappa }_1 \propto m^0 = \text{constant}$; and for a quadratic drag force, $\hat{\kappa }_1 \propto m^{2/3}$ (see Supplementary Information for derivation). It follows at once that $G_{\text{opt}}$ varies systemically with size, and that the details of this variation depend on the physical environment. Thus, and as is increasingly recognized for other musculoskeletal “design” elements such as the stiffness of in-series elastic elements (Galantis and Woledge 2003; Lichtwark and Wilson 2005, 2008; Rosario et al. 2016; Ilton et al. 2018; Mendoza and Azizi 2021), optimal gearing requires tuning to other physical and anatomical properties (Olberding et al. 2019). Is there any evidence that selection has tuned *G* to size and environment, as required for the optimization of mechanical energy flow?

**Fig. 6:**
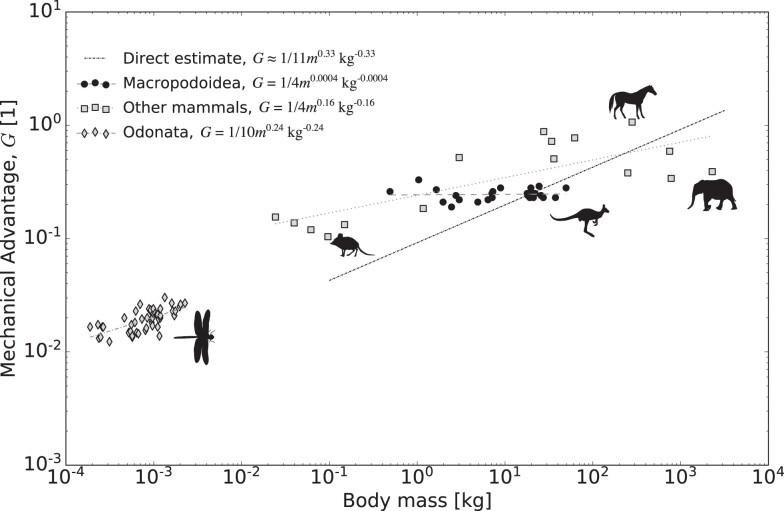
The optimal mechanical advantage is determined by two dimensionless numbers: the ungeared physiological similarity index, $\Gamma _1$, which encodes the competition between the muscle’s kinetic energy and work capacity, and the reduced parasitic energy, $\hat{\kappa }_1$, which encodes the relevance of external forces compared to the driving force. Because both numbers vary with body mass *m* under the parsimonious assumptions of isogeometry and isophysiology, so does the optimal mechanical advantage. For a contraction against gravitational force, the inertial and parasitic limit yield the same scaling coefficient, $G_{\rm opt} = \sqrt{\Gamma _1} \propto m^{1/3}$ vs $G = \hat{\kappa }_{1, g} \propto m^{1/3}$, respectively. As a test of this prediction, we directly calculated the magnitude of this optimal mechanical advantage for quadrupeds (dark dotted line), and compared it to the result of an ordinary least squares (OLS) regression on the effective mechanical advantage as measured for 16 species covering ∼6 orders of magnitude in body mass (see Biewener 1989b, 2005; Ren et al. 2010; Basu and Hutchinson 2022; and Supplementary Information for details). For a contraction against a quadratic drag force, the scaling coefficient for $G_{\rm opt}$ should fall between the parasitic limit, $G_{\rm opt} = ({\kappa _{\text{Qmax}, 1}})^{1/3} \propto m^{2/9}$, and the inertial limit $G_{\rm opt} = \sqrt{\Gamma _1} \propto m^{1/3}$ (see Supplementary Information). As a test of this prediction, we extracted the mechanical advantage of the basalar flight muscle for eight dragonfly species varying by more than one order of magnitude in body mass (see Schilder and Marden 2004 and Supplementary Information for details). OLS regression yielded a slope of 0.24, in excellent agreement with the theoretical estimate. Note that *G* can also be independent of size, as in Macropodoidea (Bennett and Taylor 1995; but see also McGowan et al. 2008).

It has long been noted that larger mammals tend to adopt a more upright posture than smaller mammals (Osborn 1900; Gregory 1912; Gray 1944). It was pointed out by Biewener (1989b) that this postural variation brings about a systematic variation in the “effective mechanical advantage” (EMA) of the limbs: crouching increases the moment arm of the ground reaction force with respect to the limb joints, and thus reduces the mechanical advantage averaged across all limb muscles. In a multi-joint system, the ratio between input and output forces cannot be reliably estimated from anatomy alone, but generally needs to be obtained through measurement of ground reaction forces. This difficulty makes EMA measurements comparatively rare; nonetheless, we extracted data for 16 mammalian quadrupeds from the literature, and estimate $\Gamma _1 \approx 0.0063 m^{2/3} \, \text{kg}^{-2/3}$ and $\hat{\kappa }_1 = 0.0123 m^{1/3} \, \text{kg}^{-1/3}$ (see Supplementary Information). Equation (15) then yields $G_{\text{opt}} \approx 1/11 \rm \space{m}^{1/3} \, \text{kg}^{-1/3}$, compared to $\text{EMA} = 1/4 m^{1/6} \, \text{kg}^{-1/6}$, estimated via an ordinary least squares regression on log10-transformed data (Fig. 6). We note that a regression restricted to Biewener’s original data yields $\text{EMA} \approx 1/4 m^{0.24} \, \text{kg}^{-0.24}$.

In notable resemblance of the scaling of EMA in terrestrial mammals, the mechanical advantage of the basalar muscle in eight species of dragonflies increases with mass as $G = 1/10 m^{0.24} \text{kg}^{-0.24}$ (data extracted from Schilder and Marden 2004, using WebPlotDigitizer; Rohatgi 2024). We were unable to extract estimates for all physical quantities required to directly predict $G_{\text{opt}}$, and thus only evaluate the scaling. Dragonflies typically fly at Reynolds numbers $\text{Re} > 10^3$ (Wakeling and Ellington 1997), so that the drag force likely varies with the square of the speed. *G* should thus scale somewhere between $G \propto m^{2/9} = m^{0.222}$ and $G \propto m^{1/3},$ in robust agreement with the empirical data, $G \propto m^{0.24}$ (see Supplementary Information and Fig. 6).

This early evidence for “tuning” is partially encouraging, and thus partially concerning. It is encouraging, for the theoretical predictions are not only in qualitative, but also in a fair degree of quantitative agreement with empirical data: the elevation for the EMA for terrestrial mammals is within a factor of three of the empirical data, and the scaling of the mechanical advantage of dragonfly flight muscle is almost identical to the prediction, and certainly within the confidence intervals (see Supplementary Information). In light of the simplicity of the analysis, and the many real features of musculoskeletal systems it ignored—muscle pennation, variations in *G* throughout the contraction, in-series elastic elements, and a plausible dependence of the maximum muscle strain on *G*—the extent of this agreement at the very least fails to reject the hypothesis that the identified mechanical constraints may hold some sway over musculoskeletal design.

However, it is also clear that a simple mechanical prediction does not tell the whole story, for the slope of EMA in terrestrial mammals deviates meaningfully from it; indeed, some animal groups, such as macropods, appear to show no size-specific variation in EMA at all (Fig. 6 and Bennett and Taylor 1995; Thornton et al. 2024, but see McGowan et al. 2008). Larger terrestrial mammals are also generally faster, and eventually slow down; both findings suggest that gearing may not be optimized to keep energy output and transmission efficiency independent of animal size (Labonte et al. 2024). Perhaps this deviation arises because some key mechanical constraints were omitted in the analysis. And perhaps some discrepancy should not be surprising altogether, for animal locomotion is constrained by more than just mechanics, and a holistic integrative perspective also ought to consider locomotor economy and neuromechanical control (Kram and Taylor 1990; Nishikawa et al. 2007; More et al. 2010; Polet and Bertram 2019; Dallmann et al. 2023; Ijspeert and Daley 2023). For now, the observation that *G* controls the flow of mechanical energy provides at least one further hypothesis not only for why larger animals should operate with larger *G* (Biewener 1989b), but also for why smaller animals should operate with smaller *G* (Usherwood 2013).

### McHenry’s objection, and the functional significance of the mechanical advantage

*This is a message [...] that challenges an assumption implicit in a century of literature. -*Matthew McHenry and Adam Summers 2011

By demonstrating that a variation in *G* is neither necessary nor sufficient to alter the maximum speed a musculoskeletal system can impart, McHenry cast doubt on the widespread practice to use *G* as a proxy for functional specialization for force vs velocity (McHenry and Summers 2011; McHenry 2012)—an objection that has been echoed by others since (Osgood et al. 2021). In the forward simulations that supported the argument, the energy input was fixed so that, without internal or external dissipative forces, force–velocity trade-offs could only ever apply instantaneously. But can muscles deliver the same energy regardless the value of *G*?

By analyzing the flow of mechanical energy during contractions with arbitrary *G*, it was demonstrated that gearing alters the musculoskeletal kinetic energy capacity, and in doing so can curtail muscle work output even in the absence of dissipative forces. It follows that there is large design space where a smaller value of *G* may well be associated with a lower force and a larger maximum output velocity—the classic force–velocity trade-off. Although there are few if any musculoskeletal systems where robust information on all relevant parameters is available, rough estimates suggest that many systems may fall into this parameter space, that is, $G > \sqrt{\Gamma }_1$ (see above and Labonte 2023; Labonte et al. 2024). Does this mean that the instantaneous perspective is salvaged, and that *G* can continue its service as a simple anatomical index for musculoskeletal specialization for force vs speed?

It was already noted above that the optimal mechanical advantage is size- and environment-dependent—this result in and of itself sends a clear warning against a functional classification of musculoskeletal systems on the basis of *G* alone. However, matters get worse still, for it will now be shown that muscle mechanical performance also depends on other geometric properties of the musculoskeletal system. In other words, even for two musculoskeletal systems that operate in the same environment, are of the same size, have the same muscle volume, and have identical muscle physiology, the magnitude of *G* does not provide a reliable indication for specialization for force vs velocity.

To put this assertion to the proof, we relate the fascicle length to the muscle volume and cross-sectional area, $L_m = V_m/A_m$, introduce the muscle aspect ratio, $\nu = L_m A_m^{-1/2}$, and rewrite Equation (19) to find


**Fig. 7:**
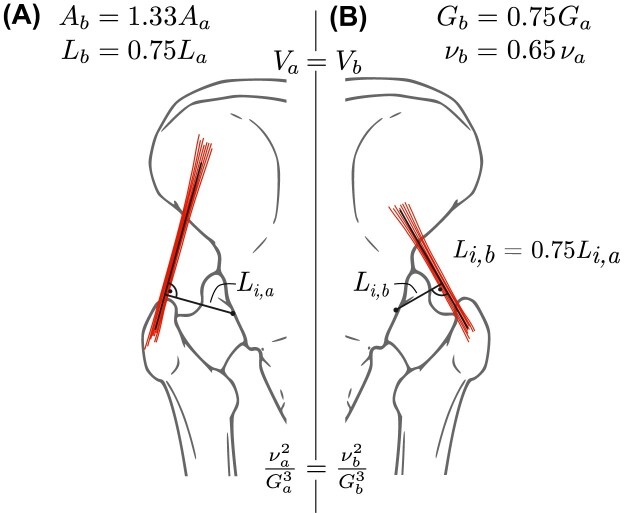
Variations of the muscle aspect ratio, $\nu = L_m A_m^{-1/2}$ can cancel the effect of variations in the mechanical advantage *G*, in the sense that musculoskeletal systems with different *G* may nevertheless operate with equal physiological similarity index $\Gamma \propto \nu ^{4/3} G^{-2}$. As an illustrative example, consider two different geometrical arrangements of the *Gluteus medius* hip abductor; muscle volume and physiology are identical, and the out-lever length (not plotted) is identical on both sides, but the in-lever length and thus mechanical advantage differs by a factor $G_a/G_b = 1.33$. As long as $\Gamma < 1$, and all else being equal, a change in *G* will result in instantaneous and ultimate force–velocity trade-offs. But it would nevertheless be premature to conclude that (**A**) is specialized for force and (**B**) is specialized for speed: the muscle in (**B**) has a larger cross-sectional area and shorter fascicle length, so that both arrangements operate with equal $\Gamma$. Because of this many-to-one mapping, a difference in mechanical advantage between study organisms cannot serve as a reliable indicator for specialization for force vs velocity by itself—neither instantaneously nor ultimately.

(21) \begin{eqnarray*}
\Gamma \propto \underbrace{\left[\frac{m}{V_m^{1/3}}\right]}_{\text{Motor density}} \overbrace{\left[\frac{\dot{\varepsilon }_{\text{max}}^2}{\varepsilon _{\text{max}} \hat{\sigma }_{\text{max}}}\right]}^{\text{Physiology}} \underbrace{\left[\frac{\nu ^{4/3}}{G^2}\right]}_{\text{Geometry}},
\end{eqnarray*}

where $\dot{\varepsilon }_{\rm max}$ is the maximum strain rate and $\hat{\sigma }_{\rm max}$ is the displacement-averaged maximum muscle stress. The right-hand side was written as the product of separate terms to highlight three distinct components of the physiological similarity index (Labonte 2023): a ratio between payload mass and motor size, muscle physiology, and musculoskeletal geometry. The motor density encodes a size-dependence for isogeometric systems, $\Gamma \propto m^{2/3}$; the physiology is parsimoniously considered conserved; but the geometry term reveals a major problem for the typical comparative interpretation of *G*: systems with different *G* may well be physiologically similar, that is, have equal $\Gamma$ (Labonte 2023), even if neither muscle physiology nor investment differ—variations in the muscle aspect ratio $\nu$ can cancel the effects of variations in *G* (Fig. 7). It is unclear why the ratio between in- and out-lever should possess a higher evolutionary flexibility than the muscle aspect ratio—both are geometric parameters defined by the ratio of two characteristic lengths. Thus, *even for a system in which a variation in *G* may modulate muscle energy output, *G* cannot be reliably interpreted as a sign for selection for force vs speed*—neither instantaneously nor in terms of mechanical energy output. Instead, robust functional interpretation requires to also consider at least the muscle aspect ratio, but $\nu$ is rarely reported or discussed in comparative studies on the mechanical advantage.

On the basis of these arguments, we join McHenry and others in their concern that an instantaneous interpretation of *G* can be functionally misleading (McHenry and Summers 2011; McHenry 2012); great care must be taken in resting the functional analysis of skeletal anatomy on a comparison of the mechanical advantage alone. Even in systems that are “overgeared,” so that instantaneous force–velocity trade-offs can map onto contraction outcomes as suggested by Arnold et al. (2011), independent variation in geometric variables such as the muscle aspect ratio can result in the mapping of many geometric designs onto the same mechanical performance: musculoskeletal systems with markedly distinct geometries can be physiologically similar (Labonte 2023). Such “many-to-one mapping” has been identified in an increasing number of mechanical systems (e.g., Arnold 1983; Alfaro et al. 2005; Strobbe et al. 2009; Blanke et al. 2018; Moen 2019; Chatar et al. 2022), and can enable morphological and physiological diversification despite a convergence in mechanical function (Wainwright et al. 2005; Wainwright 2007; Muñoz et al. 2018; Muñoz 2019). Viewed in this light, our warning against an isolated interpretation of *G* is not a pessimistic negation of the functional significance of *G*, but an optimistic proposal to re-evaluate the anatomical diversity of musculoskeletal anatomy through the functional interpretation derived from the theory of physiological similarity.

### Outlook

A mechanical analysis of a minimalist musculoskeletal system suggested a physical interpretation of the mechanical advantage that is robustly rooted in the conservation of mechanical energy. Illustrative applications of this framework revealed promising qualitative and semi-quantitative agreement between theoretical predictions and observable features of musculoskeletal systems. However, this initial success must not be allowed to mask the fact that several essential aspects that add further complexity were omitted: the mechanical advantage does not remain constant throughout a contraction (Carrier et al. 1994, 1998; Olberding et al. 2019); elastic elements such as tendons, apodemes, and aponeuroses may obscure the relationship between muscle input and skeletal output (Galantis and Woledge 2003; Eng and Roberts 2018; Olberding et al. 2019); we only provided a cursory treatment of the effect of force–velocity properties, and neglected force–length properties; many muscles are pennate, and the pennation angle thus provides another architectural degree of freedom; gearing due to pennation can depend on the magnitude of the applied muscle force (Azizi et al. 2008; Eng et al. 2018); and this relation likely varies with other key biological variables such as age (Holt et al. 2016). Without doubt, more complex and accurate mechanical analyses are in order to put the merits of the theory of physiological similarity to a rigorous test; the problem requires further attention, and many details remain unresolved.

The combination of mathematical simplicity, experimental accessibility, and functional consequence has rendered *G* a rather attractive anatomical metric. These are no doubt desirable qualities of scientific theories, but perhaps an exclusively instantaneous interpretation is so simple that it risks to blind us to other relevant functional implications of variations in *G*. Analyzing the effects of gearing in terms of mechanical energy permits a more holistic analysis of the functional and comparative anatomy of musculoskeletal systems than an instantaneous perspective affords, and brings about the exciting opportunity to integrate mechanical analyses with powerful evolutionary concepts such as many-to-one mapping, morphological modularity, and integration. Such future work holds significant promise to progress our understanding of the evolutionary biomechanics of animal movement.

## Supplementary Material

icae072_Supplemental_File

## Data Availability

All data are incorporated into the article and its online supplementary material.

## Notes

From the symposium “Modeling Organismal Responses to Changing Environments” presented at the annual meeting of the Society for Integrative and Comparative Biology, January 2–6, 2024.
