# Supplementary material for: Optimal Gearing of Musculoskeletal Systems
Source: Integr Comp Biol. 2024 Jun 20;64(3):987–1006. doi: 10.1093/icb/icae072 (PMC11445786; doi:10.1093/icb/icae072)
Supplement: icae072_Supplemental_File [file icae072_supplemental_file.pdf]

# Supplementary Information for: Optimal Gearing of Musculoskeletal Systems

D. T. Polet and D. Labonte 2024
Original article: https://doi.org/10.1093/icb/icae072

## Optimal gearing for velocity-dependent external forces

### *General procedure*

The external force $F_e$ may depend on the velocity, $F_e(u)$. A common biological example of velocity-dependent external force is the drag force, $F_D$. Drag is a complex physical phenomenon, and a first-order model typically considers a drag force that varies either linearly or quadratically with speed. A linear drag force, $F_D \propto u$, is an appropriate model for viscous drag, which dominates at small Reynolds numbers (that is, slow speed, high viscosity, or small length). At intermediate to large Reynolds numbers, a quadratic drag force, $F_D \propto u^2$, is more appropriate.

The general analysis for both a linear and quadratic drag force proceeds from the conservation of energy:

$$\begin{aligned} W =& \Delta K \\ \int (F_o - F_D)\, \mathrm{d}x =& m \int u\, \mathrm{d}u \end{aligned} \tag{S1}$$

Because the drag force depends on $u$, solution requires separation of variables:

$$\int F_o\, \mathrm{d}x = m \int \frac{u}{1 - F_D/F_o}\, \mathrm{d}u \tag{S2}$$

To introduce the mechanical advantage, we use the couplings $F_0 = F_i G$, and $x = \delta/G$, and note that $G$ is optimal if the maximum displacement and shortening speed of muscle are reached simultaneously, which defines the upper integration boundary for both the displacement- and the velocity-integral, and thus yields:

$$\int^{\delta_{\max}} F_i\, \mathrm{d}\delta = m \int^{u_{max}} \frac{u}{1 - F_D/(F_i G_{\mathrm{opt}})}\, \mathrm{d}u \tag{S3}$$

For a linear drag force, $F_{D,L} = \beta_L u$, with $\beta_L$ as linear drag multiplier, the velocity integral evaluates to:

$$\begin{aligned} W_{\max} =& m \int_0^{u_{\max}} \frac{u}{1 - u\,\beta_L/(F_i G_{\mathrm{opt}})}\, \mathrm{d}u \\ \frac{\kappa_{\max,1}}{\Gamma_1} =& -2\left[1 + \frac{G_{\mathrm{opt}}^2}{\kappa_{\max,1}} \log\left(1 - \frac{\kappa_{\max,1}}{G_{opt}^2}\right)\right] \end{aligned} \tag{S4}$$

where we used the coupling $u = v/G$ and introduced $\kappa_{\max,1} = \beta_L v_{\max} F_{\max}^{-1}$ as the ratio of the maximum drag and driving force for an ungeared muscle (i. e. a mechanical advantage of unity).

For a quadratic drag force, $F_{D,Q} = \beta_Q u^2$, with $\beta_Q$ as quadratic drag multiplier, one finds instead:

$$\begin{aligned} W_{\max} =& m \int_0^{u_{\max}} \frac{u}{1 - u^2\,\beta_Q/(F_i G_{\mathrm{opt}})}\, \mathrm{d}u \\ \frac{\kappa_{\max,1}}{\Gamma_1} =& -G_{\mathrm{opt}} \log\left(1 - \frac{\kappa_{\max,1}}{G_{opt}^3}\right) \end{aligned} \tag{S5}$$

where $\kappa_{\max,1} = \beta_Q v_{\max}^2 F_{\max}^{-1}$ has the same physical meaning as before.

Thus, for both a linear and a quadratic drag force, the optimal mechanical advantage is determined uniquely by two dimensionless numbers, $\Gamma_1$ and $\kappa_{\max,1}$.

### *The optimal mechanical advantage for linear drag*

For the linear drag force, an explicit solution for $G_{opt}$ can be found:

$$G_{\mathrm{opt}} = \frac{\sqrt{\kappa_{\max,1}}\sqrt{\frac{\kappa_{\max,1}}{2\Gamma_1} + 1}}{\sqrt{\frac{\kappa_{\max,1}}{2\Gamma_1} + 1 + \mathrm{W}\left[-\exp\left(-\frac{\kappa_{\max,1}}{2\Gamma_1} - 1\right)\left(1 + \frac{\kappa_{\max,1}}{2\Gamma_1}\right)\right]}} \tag{S6}$$

where $W$ is the Lambert W function. It is hard to develop an intuitive feel for this expression, but the limits provide a clear physical picture. For convenience, we define $\gamma = \kappa_{\max,1}/(2\Gamma_1)$, and consider the limits $\gamma \to 0$ (inertial forces dominate) and $\gamma \to \infty$ (external force dominate). Through Taylor expansion about $\gamma = 0$, we find $\lim_{\gamma\to 0}\left[1 + \mathrm{W}\left(-\exp\left(-\gamma - 1\right)\left(1 + \gamma\right)\right)\right] = \gamma$. The right-side limit for the remaining term is:

$$\lim_{\gamma\to 0} G_{\mathrm{opt}} = \sqrt{\Gamma_1} \tag{S7}$$

In this limit, inertial forces dominate, and the optimal mechanical advantage is thus independent of external forces. For $\gamma \to \infty$, the productlog-term goes to 0, which yields:

$$\lim_{\gamma\to\infty} G_{\mathrm{opt}} = \sqrt{\kappa_{\max,1}} \tag{S8}$$

The inertial force is now irrelevant, and the optimal mechanical advantage is the value of $G$ which ensures a dynamic equilibrium of the parasitic and driving force.

For any intermediate value of $\gamma$, the optimal gear ratio depends on a complex combination of the three key forces: the inertial force, the driving force, and the parasitic force, and is defined exactly by Eq. S6.

### *The optimal mechanical advantage for quadratic drag*

To the best of our judgement, the implicit expression for $G_{\mathrm{opt}}$ allows no explicit writing for a quadratic drag force; $G_{\mathrm{opt}}$ has to be determined numerically. However, the limits can still be assessed, and follow in direct analogy to the solution for a linear drag force.

When the drag force is large, $\gamma = \kappa_{Q\max,1}/\Gamma_1 \to \infty$, and Eq. S5 yields:

$$\lim_{\gamma\to\infty} G_{\mathrm{opt}} = \left(\frac{\beta_Q v_{\max}^2}{F_{\max}}\right)^{1/3} = \sqrt[3]{\kappa_{Q\max,1}} \tag{S9}$$

which is equivalent to equilibrium of maximum dynamic forces (see also Richards and Clemente 2013):

$$\begin{aligned} F_{\max,o} =& F_{\max,D} \\ G_{\mathrm{opt}} F_{\max} =& \beta_Q u_{\max}^2 \\ G_{\mathrm{opt}} =& \left(\frac{\beta_Q v_{\max}^2}{F_{\max}}\right)^{1/3} \end{aligned} \tag{S10}$$

When inertial forces dominate, $G_{\mathrm{opt}} \to \sqrt{\Gamma_1}$, which follows as before.

Recognizing that both quadratic and linear drag have the same inertial limit for $G_{\mathrm{opt}}$, the symbolic results for linear drag above can be used to approximate quadratic drag by setting the upper $G_{\mathrm{opt}}$ limits to be equal. This implies:

$$\beta_{\mathrm{L,approx}} = \sqrt[3]{\beta_Q^2 v_{\max} F_{\max}} \tag{S11}$$

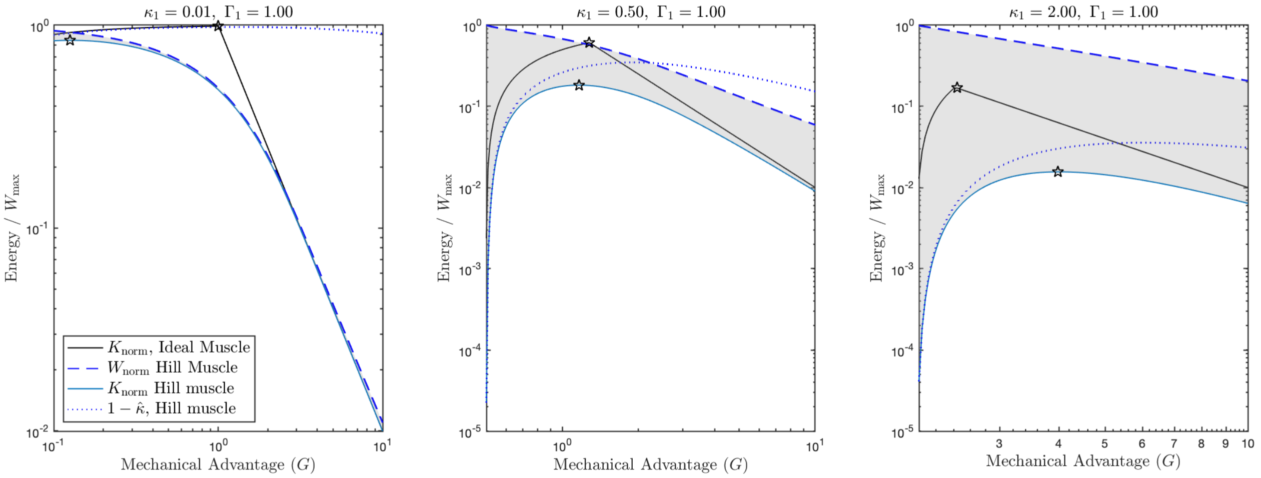


**Fig. S1.** The energy landscapes for a Hill-muscle with a linear force-velocity relationship are qualitatively similar to those for an idealised muscle; the examples shown here are for contractions against a constant external force. From left to right, the ratio of external to muscle force $\kappa_1$ increases; for all cases, $\Gamma_1 = 1$. For both a Hill- and an idealised muscle, the work output (dashed line) decreases as $G$ is increased. Reducing $G$ by too much, in turn, reduces the fraction of work that flows into kinetic energy, the transmission efficiency $\eta = K/W_m = 1 - \hat{\kappa}$, dotted line), and increases the time required to deliver each unit of work (not shown). Thus, although the mechanical advantage that maximizes kinetic energy output will now have a different magnitude, it will still be intermediate. This result arises, because the energy outputs of Hill-muscle (blue solid line) and the idealised muscle (black solid line) are indistinguishable in the limits of vanishing and diverging $G$, respectively (Labonte 2023).

## Energy flow of geared muscle with force-velocity properties

Real muscle has more complex force-velocity properties than the idealised muscle studied in most of this work: the force it can exert is sub-maximal for any shortening speed larger than zero. For a "Hill-muscle", the force varies between a maximum $F_i(v = 0) = F_{\max}$ in an isometric contraction, and a minimum $F_i(v = v_{\max}) = 0$ during a contraction with maximum speed. The dynamics become more complex, but the energy landscape can still be determined from the corresponding equations of motion (Labonte 2023). The quantitative appearance of the energy landscapes clearly differs between an idealised and a Hill-muscle (Supplemental Fig.S1), but the qualitative picture remains unchanged: muscle work output vanishes as $G$ diverges; and as $G$ vanishes, delivering the same amount of work takes more time, and the fraction that flows into kinetic energy (the transmission efficiency $\eta = K/W_m = 1 - \hat{\kappa}$) approaches zero–the same trade-offs exist, and the optimal mechanical advantage will be of intermediate magnitude. This result arises, because the idealised and the Hill-muscle become indistinguishable in the limits of a vanishing and diverging $G$ (see Labonte 2023, for further discussion).

## The allometry of the mechanical advantage

The "effective mechanical advantage" that characterises the limbs of terrestrial mammals was extracted for 16 quadruped and 24 macropod species from Biewener (1989); Bennett and Taylor (1995); Biewener (2005); Ren et al. (2010); Basu and Hutchinson (2022), as described in Labonte et al. (2024). Data on the mechanical advantage of the basalar muscle of the dragonfly flight apparatus was extracted from Schilder and Marden (2004), using Ankit Rohatgi's WebPlotDigitizer (Rohatgi 2024). This paper reports in-lever, out-lever and basalar muscle mass for 47 individuals from eight species, but does not contain an explicit relationship between body mass and mechanical advantage. In order to obtain this relationship, basalar muscle mass and body mass were related via ordinary least square (OLS) regression of log10-transformed data. Mean estimates for the basalar muscle mass $m_m$ for each species were extracted from Fig. 4, and the mean body mass $m_b$ was provided in Tab. 1. OLS regression then yielded the relation $m_b = 71m_m$. In-lever and out-lever were extracted from Fig. 5A and B, and lever pairs formed as accurately as possible, using the magnitude of the basalar muscle mass and species identification as guides.

The relationship between body mass and mechanical advantage is influenced not only by mechanical and ecological constraints, but also by evolutionary history (Biewener 2005). *In lieu* of a phylogenetic regression, we roughly estimated the strength of the phylogenetic constraints by conducting four separate OLS regressions on log10-transformed data, summarised in Tab. 1: one including all data, one only for dragonflies, one only for macropods, and one for all mammalian quadrupeds. In order to directly estimate the optimal mechanical advantage for terrestrial mammals, we estimate $\Gamma_1 \approx 0.0063\text{mass}^{2/3}\text{kg}^{-2/3}$ and $\hat{\kappa}_1 = 0.0123\text{mass}^{1/3}\text{kg}^{-1/3}$ for the gravitational force, using the data provided in in Tab. 1 in Labonte et al. (2024). Eq. 15 (main manuscript) then yields $G_{\text{opt}} \approx 1/11\text{mass}^{1/3}\text{kg}^{-1/3}$.

The optimal mechanical advantage of the dragonfly flight muscle should fall somewhere between the inertial and force-balance limit. The inertial limit gives $G_{\text{opt}} = \sqrt{\Gamma_1}$. As $\Gamma_1 \propto m^{2/3}$, $G_{\text{opt}} \propto m^{1/3}$ in the inertial limit. Balance of aerodynamic forces yields a prediction $G_{\text{opt}} = \left(\frac{\beta_Q v_{\max}^2}{F_{\max}}\right)^{1/3}$, where $\beta_Q$ is a quadratic drag multiplier (proportional to area). Using $\beta_Q \propto m^{2/3}$, $F_{\max} \propto m^{2/3}$ and $v_{\max} \propto m^{1/3}$, predicts $G_{\text{opt}} \propto m^{2/9}$. For a linear drag force, one finds $G_{\text{opt}} = \sqrt{\kappa_{\max,1}} = \sqrt{\beta_L v_{\max} F_{\max}^{-1}}$. Isogeometry and isophysiology imply $\beta_L \propto \text{length} \propto m^{1/3}$, and $F_{\max} \propto m^{2/3}$, so that $G_{\text{opt}} \propto m^0 = \text{constant}$. Note well that this differs from the typical prediction of a drag force that is relatively larger for smaller individuals; this discrepancy arises because we note that the maximum velocity itself is also size-dependent, provided that

**Table 1.** Results of ordinary least squares regression on log10-transformed data, with body mass in kilograms. Values in parentheses indicate 95% confidence intervals.

| | Elevation | Slope | $R^2$ |
|---|---|---|---|
| All | 0.14 [0.13; 0.16] | 0.27 [0.25; 0.29] | 0.93 |
| Odonata | 0.1 [0.06; 0.19] | 0.24 [0.16; 0.32] | 0.44 |
| Macropodoidea | 0.25 [0.22; 0.27] | 0.004 [-0.04; 0.05] | 0.002 |
| Other mammals | 0.24 [0.18; 0.33] | 0.16 [0.08; 0.23] | 0.61 |

the musculoskeletal system is limited by its shortening velocity Labonte (2023).

## The magnitude of the optimal mechanical advantage for an inertial contraction

If muscle provides the dominant force in the system, the contraction is approximately inertial, and the magnitude of the optimal mechanical advantage follows as:

$$G_{\text{opt}}^2 = \frac{K_{\max}}{W_{\max}} = \frac{1}{2} L_m^2 \frac{\dot{\varepsilon}_{\max}^2}{W_\rho} m_f \tag{S12}$$

where $W_\rho \approx 70\,\text{J}\,\text{kg}^{-1}$ is the work density of muscle, and $m_f = m_m m^{-1}$ is the ratio between muscle mass and payload mass (Labonte et al. 2024). For a representative maximum strain rate $\dot{\varepsilon}_{\max} = 10$ lengths per second, and a plausible value of $m_f = 0.1$, one may find:

$$G_{\text{opt}} \approx \frac{1}{4} L_m \text{meter}^-1 \tag{S13}$$

which is the estimate used in the main manuscript.

## Case studies

### *Praying mantis strike*

The predatory strike of the raptorial forelimb of a praying mantis (*Heirodula membranacea*) provides an example of a rapid, approximately inertial movement. To determine the optimal mechanical advantage, only the ungeared physiological similarity index $\Gamma_1$ needs be estimated.

Gray and Mill (1983) point to the parallel-fibred thoracic trochanteral extensor (TTrE) and pennate coxal trochanteral extensor (CTrE) and as the primary extensors of the coxo-trochanteral joint. These muscle have a combined mass of 29.6 mg, yielding a work capacity of $W_{\max} = 2.1$ mJ assuming a work density of 70 J/kg (Labonte et al. 2024). As these muscles have differential fiber lengths, a single "effective" fiber length must be calculated as $W_{\max}/(\sum F_{i,\max})/\varepsilon_{\max}$, where $\varepsilon_{\max}$ is the maximum strain rate (taken as 0.3, Labonte et al. 2024), and muscle forces are summed.

Gray and Mill (1983) list the muscle area as 1.5 and 3.5 $\text{mm}^2$ for the TTrE and CTrE respectively, with a maximum pennation angle of 20.3° for the CTrE. With a maximum stress of 250 kPa (following Labonte et al. 2024), this yields a summed force of 1.2 N, and thus an effective fiber length of 5.8 mm. Note that this is between the reported fiber lengths of 10.4 and 4.1 mm of the TTrE and CTrE respectively (Gray and Mill 1983). For these muscles, Gray and Mill (1983) report a maximum strain rate of 15 $\text{s}^{-1}$.

To determine the moment of inertia of the forelimb distal to the coxa, the femur and trochanter were modeled as an ellipsoid, while the tibia was modeled as a cylinder, each with constant mass density of 1 $\text{g/cm}^3$. Length dimensions were extracted from figure 6 in Gray and Mill (1983), with the same pose assumed, and out-of-plane thickness of the femur was taken as 1/9 femur+trochanter length. We neglect the inertia of the tarsus. This yielded a combined mass of 0.17 g, and radius of gyration of 1.85 cm.

Combination of these estimates results in $\Gamma_1 = 3.1 \times 10^{-4}$, and thus $G_{\text{opt}} = \sqrt{\Gamma_1} = 0.018$. Gray and Mill (1983) report coxo-trochanteral moment arms between 0.32 to 1.07 mm during the strike; divided by the radius of gyration, this corresponds to $G_{\text{emp}} \in [0.017, 0.058]$.

### *Kangaroo rat jump*

The jump of a kangaroo rat serves as an example for a contraction against non-negligble gravitational forces. Rankin et al. (2018) present a musculoskeletal model of *Dipodomys deserti* from which relevant parameters can be obtained. The animal has a body mass 106 g and three ankle extensors per leg (Plantaris and lateral and medial Gastrocnemius) with combined mass, work capacity and maximum force of 2.63 g, 0.184 J and 68.8 N (summed across two legs). This yields an effective fiber length of 18 mm. Javidi et al. (2020) report a maximum contraction velocity of 12 fiber lengths per second, which then yields $\Gamma_1 = 0.013$. Assuming the dominant external force is body weight, and using a gravitational acceleration of 9.81 $\text{m}\,\text{s}^{-2}$, yields $\hat{\kappa}_1 = 0.015$.

Rankin et al. (2018) report maximum ankle extension moments of 146 to 167 N mm at 90 ankle flexion. This corresponds to in-lever moment arms of 4.24 to 4.85 mm, close to the values of 5.6 mm reported by Biewener and Blickhan (1988). For the outlever length, we assume the ground reaction force vector to be vertical with a center of pressure somewhere along the phalanges. Using model data presented by Rankin et al. (2018), the maximum outlever length was defined as (midfoot + toes) - (min in-lever), and the minimum outlever was defined as (midfoot - max in-lever). With a midfoot length of 24 mm and a toe length of 17.9 mm, this yields an empirical estimate of $G_{\text{emp}} \in [0.11, 0.24]$.

### *Frog swimming*

As an example for contractions against a drag force, we study the plantaris-driven ankle extension of *Xenopus laevis*, following Richards (2008) and Richards and Sawicki (2012). From the latter study, we extract a frog of body mass of 30 g and a foot mass of 3 g. With this body mass, we use regression equations from Clemente and Richards (2013) to determine muscle length ($l_m = 2.1$ cm), foot area ($A = 5.7\ \text{cm}^2$), foot length (2.8 cm), maximum isometric force ($F_{\max} = 7.0$ N), and maximum contraction velocity ($v_{\max,rel} = 6.8$ lengths per second). The work capacity follows as the product between isometric force times muscle length with a maximum strain of 0.3 (Labonte et al. 2024), giving 0.0445 J. Using the trapezoidal approximation of foot area and length measurements from Richards (2008), and assuming a constant mass density, we calculate the moment of inertia of such a foot about the ankle as $I = 0.01\ \text{kg}\,\text{cm}^2$, and a center of area at $R = 1.6$ cm from the ankle. This gives a "mass equivalent" of $I/R^2 = 4$ g.

The ungeared physiological similarity index is therefore $\Gamma_1 = 8.7 \times 10^{-4}$.

Following Richards and Clemente (2013) we use a drag coefficient of $C_d = 2$ for the foot, and determine the quadratic drag multiplier $\beta_Q = 1/2\rho A C_d = 0.57$ kg m$^{-1}$, where $\rho = 1000$ kg m$^{-3}$ is the density of water. The equivalent linear drag multiplier is thus $\beta_L = 0.69$ kg s$^{-1}$ (Eq. S11). The maximum ungeared force ratio for linear drag follows as $\kappa_{\max,1} = \beta_L l_m v_{\max,rel}/F_{\max} = 0.014$.

The plantaris moment arm is also derived from a regression equation (Clemente and Richards 2013) as 0.16 cm. Compared to the center of area, this yields a mechanical advantage of 0.1. A lower mechanical advantage of 0.06 is reported by Richards and Clemente (2013) in *Xenopus laevis*. These two values are taken as a sensible empirical range. We thus estimate $G_{\text{emp}} \in [0.06, 0.1]$

## References

Basu C, Hutchinson JR. 2022. Low effective mechanical advantage of giraffes limbs during walking reveals trade-off between limb length and locomotor performance. Proceedings of the National Academy of Sciences 119:e2108471119. doi:10.1073/pnas.2108471119.

Bennett MB, Taylor GC. 1995. Scaling of elastic strain energy in kangaroos and the benefits of being big. Nature 378:56–9.

Biewener AA. 1989. Scaling body support in mammals: limb posture and muscle mechanics. Science 245:45–48.

Biewener AA. 2005. Biomechanical consequences of scaling. J Exp Biol 208:1665–1676.

Biewener AA, Blickhan R. 1988. Kangaroo rat locomotion: design for elastic energy storage or acceleration? Journal of Experimental Biology 140:243–255.

Clemente CJ, Richards C. 2013. Muscle function and hydrodynamics limit power and speed in swimming frogs. Nature Communications 4:2737. doi:10.1038/ncomms3737.

Gray PTA, Mill PJ. 1983. The mechanics of the predatory strike of the praying mantid *Heirodula Membranacea*. Journal of Experimental Biology 107:245–275. doi:10.1242/jeb.107.1.245.

Javidi M, McGowan CP, Lin DC. 2020. Estimation of the forcevelocity properties of individual muscles from measurement of the combined plantarflexor properties. Journal of Experimental Biology 223:jeb219980. doi:10.1242/jeb.219980.

Labonte D. 2023. A theory of physiological similarity in muscle-driven motion. Proceedings of the National Academy of Sciences 120:e2221217120. doi:10.1073/pnas.2221217120.

Labonte D, Bishop PJ, Dick TJM, Clemente CJ. 2024. Dynamic similarity and the peculiar allometry of maximum running speed. Nature Communications 15:2181. doi:10.1038/s41467-024-46269-w.

Rankin JW, Doney KM, McGowan CP. 2018. Functional capacity of kangaroo rat hindlimbs: adaptations for locomotor performance. Journal of the Royal Society Interface 15:20180303. doi:10.1098/rsif.2018.0303.

Ren L, Miller CE, Lair R, Hutchinson JR. 2010. Integration of biomechanical compliance, leverage, and power in elephant limbs. Proceedings of the National Academy of Sciences 107:7078–7082. doi:10.1073/pnas.0911396107.

Richards CT. 2008. The kinematic determinants of anuran swimming performance: an inverse and forward dynamics approach. Journal of Experimental Biology 211:3181–3194. doi:10.1242/jeb.019844.

Richards CT, Clemente CJ. 2013. Built for rowing: frog muscle is tuned to limb morphology to power swimming. Journal of The Royal Society Interface 10:20130236. doi:10.1098/rsif.2013.0236.

Richards CT, Sawicki GS. 2012. Elastic recoil can either amplify or attenuate muscletendon power, depending on inertial vs. fluid dynamic loading. Journal of Theoretical Biology 313:68–78. doi:10.1016/j.jtbi.2012.07.033.

Rohatgi A. 2024. WebPlotDigitizer v 4. https://automaris.io

Schilder RJ, Marden JH. 2004. A hierarchical analysis of the scaling of force and power production by dragonfly flight motors. J. Exp. Biol. 207:767.
